# Brick Strex: a robust device built of LEGO bricks for mechanical manipulation of cells

**DOI:** 10.1038/s41598-021-97900-5

**Published:** 2021-09-16

**Authors:** Elina Mäntylä, Teemu O. Ihalainen

**Affiliations:** BioMediTech, Faculty of Medicine and Health Technology, Tampere University, Arvo Ylpön katu 34, 33520 Tampere, Finland

**Keywords:** Fluorescence imaging, Biophysical methods, Cell signalling, Cellular imaging, Biomedical engineering

## Abstract

Cellular forces, mechanics and other physical factors are important co-regulators of normal cell and tissue physiology. These cues are often misregulated in diseases such as cancer, where altered tissue mechanics contribute to the disease progression. Furthermore, intercellular tensile and compressive force-related signaling is highlighted in collective cell behavior during development. However, the mechanistic understanding on the role of physical forces in regulation of cellular physiology, including gene expression and signaling, is still lacking. This is partly because studies on the molecular mechanisms of force transmission require easily controllable experimental designs. These approaches should enable both easy mechanical manipulation of cells and, importantly, readouts ranging from microscopy imaging to biochemical assays. To achieve a robust solution for mechanical manipulation of cells, we developed devices built of LEGO bricks allowing manual, motorized and/or cyclic cell stretching and compression studies. By using these devices, we show that $\upbeta$-catenin responds differentially to epithelial monolayer stretching and lateral compression, either localizing more to the cell nuclei or cell–cell junctions, respectively. In addition, we show that epithelial compression drives cytoplasmic retention and phosphorylation of transcription coregulator YAP1. We provide a complete part listing and video assembly instructions, allowing other researchers to build and use the devices in cellular mechanics-related studies.

## Introduction

Cells in our bodies form a complex biochemical and mechanical homeostasis with the surrounding tissues^1,2^. In addition to biochemical signals, cellular physiology is influenced by various physical factors of extracellular environment (topography, rigidity, cell ligand density) and mechanical forces (tensile, shear or compressive forces)^3–5^. Cells can sense physical cues via mechanotransduction process, where mechanical signals are converted into biochemical or electrical activity of the cells. Mechanotransduction events often take place in the cell extracellular matrix (ECM) and cell–cell adhesions, which both contain mechanosensitive proteins. This allows the cells to transform the mechanical signals into intracellular signaling and to adjust the physiological processes accordingly^2,6^. Mechanotransduction is achieved when the mechanosensitive proteins respond to mechanical force, expose cryptic binding sites, and trigger downstream signaling cascades such as co-operating Wnt/$\upbeta$-catenin and Hippo pathways or YAP/TAZ signaling^7–11^.

In addition to cell–cell and cell-ECM junctions, mechanical forces are also sensed deeper within the cells, and they have been shown to directly affect nuclear lamina, chromatin, transcription, and genetic programming^12–16^. Via these signaling pathways, mechanical forces co-regulate various cellular processes including proliferation, differentiation and cell migration allowing cells to probe the environment and to adjust their physical properties^13,17,18^. Different cell types encounter a variety of mechanical cues: endothelial cells are subjected to shear stress from the blood flow, muscle cells contract and produce strong contractile forces, and epithelial cells experience tension, stretching and compression^19^. Mechanical forces are especially important for normal epithelial homeostasis, and they are often altered in diseases such as cancer, where cellular contractility is modified, and higher compressive forces rise from the confined growth of the tumor^20,21^. Furthermore, intercellular contact-induced arrest of cell growth underlies the homeostatic control of cell growth, density, and tissue/organ size^7,22^. In recent years, studies on mechanical stretching have been uncovering the cellular responses to strain^23–26^. However, the opposite phenomenon, lateral compression, has remained understudied despite its importance for e.g., muscle function and implications in cancer.

Studying the effects of mechanical stimuli on cells requires replicable experiments conducted under well controlled in vitro conditions with high throughput enabling both microscopy and biochemical analyses. Until now, several approaches have been designed to cover these demands. Still, many of the developed devices need special techniques and are most often limited by the small number of cells. In these approaches, single cell or even subcellular mechanical manipulation is conducted via micromanipulation or by atomic force microscopy^27–29^. However, different and more robust approaches have been developed for larger cell populations. Mechanical compressive stress has been imposed on cells by using various gel- or glass coverslip -based compression schemes^30,31^. Tensile forces have also been imposed to cell populations via different stretching devices, where an elastic cell culture substrate is stretched mechanically or pneumatically^32–35^. Many of these devices function uniaxially, compress cell culture vertically, or apply biaxial strain^36–40^. Current market leaders distribute devices with polydimethylsiloxane (PDMS) stretch chambers with actuators for stretch applications including the Nepagene, CellScale and StrexCell^41^. These devices can be excellent for the reproducibility of the experiments but might include downsides. In addition to price, the dynamic range of the movements can be limited, or the size of the devices and the number of cells used do not allow biochemical analyzes preceded by cell fractionation. Here, we describe simple, cost-effective and robust cell stretching devices, Brick Strex S and Brick Strex L, built entirely of LEGO bricks. These bricks were chosen due to their dimensional precision, low cost, durability and sterilization possibilities. These devices allow uniaxial stretching or compression of large cell populations. The systems can be used manually, or they can be combined with motors of LEGO Mindstorms, allowing automation of the stretching experiments. We used the Brick Strex S and L systems for mechanical stimulation of epithelial cells by lateral mechanical compression and thorough immunohistochemical analyses of the cellular responses therein. We envision that the described systems provide an easy starting point for mechanical manipulation of cells and microtissues both in fundamental research and in teaching.

In this article, we provide stepwise assembly instructions, parts listing, and videos for building the systems. We show that the systems are simple, inexpensive, flexible and applicable with biochemical analyses including immunostaining, cell fractioning, Western blot, and even real-time live-cell confocal microscopy imaging. We present the functionality of our system in studies of mechanobiological phenomena and illustrate that the increased lateral compression of epithelial cells leads to concurrent increase in cell density, reduced cell size, relocalization of $\upbeta$-catenin to the cell vertexes including cell–cell junctions and increased cytoplasmic localization and phosphorylation of transcriptional regulator yes-associated protein 1 (YAP1). Thus, the here introduced Brick Strex systems are cost-effective and versatile cell manipulation systems with high reproducibility enabling mechanical manipulation of the cells with broad implications on the fields of tissue homeostasis and disease pathogenesis.

## Results

### Construction of Brick Strex S and Brick Strex L devices

**Figure 1:**
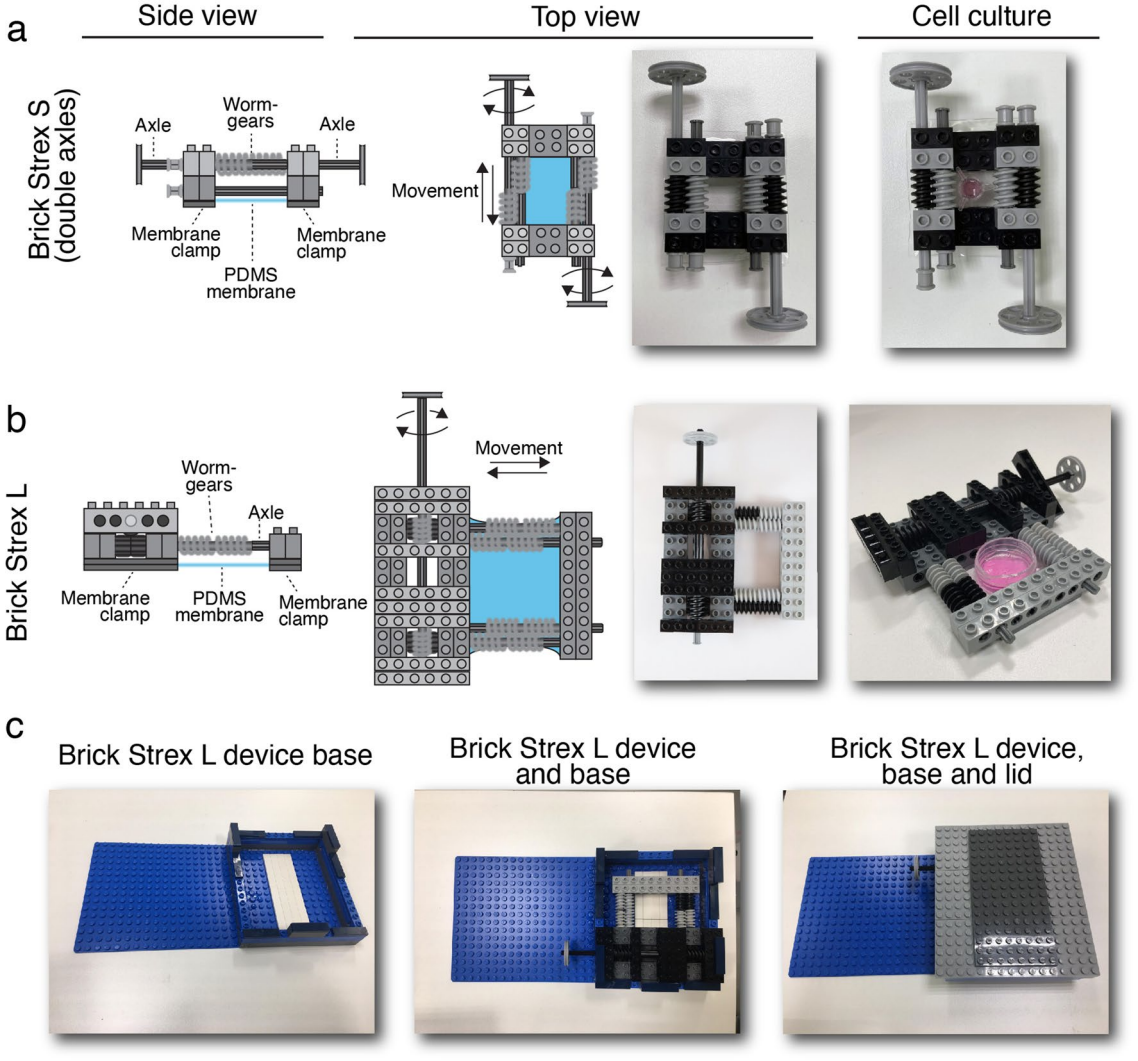
Brick Strex devices and their structure. Design of the two Brick Strex cell stretching devices built of LEGO bricks. (**a**) Side and top views of Brick Strex S device (left and middle panels). (**b**) Side and top views of Brick Strex L device. Both devices can be easily combined to cell culture with or without a cell culture well attached to the membrane (a and b, right panels). (**c**) Brick Strex L device base alone (lower left panel), combined with the device (lower middle panel) and with the lid attached (lower right panel).

The smaller Brick Strex S device is built of 34 parts (or 24 parts if only one axle is used) to create a robust core, membrane clamps, and axles for tuning the stretch (Fig. 1a,). For a comfortable and easy start, we provide a list of parts and complete assembly figures and videos (Supplementary Table 1, Supplementary Fig. 1 and Supplementary Movie 1). The device is ~ 5 cm wide, ~ 8 cm long and ~ 1.5 cm high enabling easy handling and maintenance in cell culture incubator on a 10 cm Petri dish. After building the core, a PDMS membrane (6 × 8 cm) was attached to the bottom of the device by always clamping it to a small pre-strain (less than 1%). This pre-strained condition was considered as the 0-strain state. The membrane was then supplemented with a plastic polycarbonate column (surface area ~ 0.3 cm^2^) cut off from a cell culture transwell insert. Column was attached to the membrane with silicone grease. However, since the device fits into a 10 cm Petri dish, it can be used also without the column to avoid limiting the cell culture area.

For the stretching experiments, membranes were protein coated with fibronectin to adapt for cell culture and finally, seeded with cells. The cells were grown for 2 days until a desired confluency. Next, the cell culture column was removed and replaced with a larger one enabling stretching of the cells without a damaging edge-effect, and the membrane was stretched to a 25% tensile strain. In principle, the applied tension in the stretching experiments leads to enlargement of the PDMS membrane surface area under the adhered cells sensing lateral stretching. This results in pulling of the cells away from each other observed as a decrease in the number of cells per field of view i.e., reduced packing density.

For the compression experiments, the cells were seeded into the transwell column attached onto a pre-stretched (25% tensile strain) membrane and kept in place during the compression since the silicone grease allows easy and smooth sliding of the column on top of the membrane. After the compression, the condition of the PDMS membrane is relaxed. Respectively, the relaxation of the membrane tension in the compression experiments lead to shrinkage of the PDMS membrane surface area under the adhered cells sensing lateral compressive forces i.e., compression. This in turn resulted in pushing of the cells towards each other observed as an increase in the number of cells per field of view i.e., increased cell packing density.

To increase the substrate surface area to facilitate higher cell numbers and to allow live cell imaging, we developed a larger Brick Strex L device. It contains a 56 -part core (containing membrane clamps and a big worm-geared axle for tuning the stretch) which is combined with a base suitable for live cell imaging in medium (31 bricks) and a lid to cover for protection if desired (20 bricks) (Fig. 1b,c). The device is ~ 11 cm wide, ~ 13 cm long and ~ 3 cm high and fits into a 15 cm Petri dish enabling convenient handling and maintenance in a cell culture incubator. After building the core, a PDMS membrane (6 × 8 cm) was attached by clamping to the bottom of the device and supplemented with 8.8 cm^2^ plastic column cut from a 50 mL NUNC-tube and attached with silicone grease (optionally, an in-house-3D-printed column can be used, data not shown) followed by fibronectin coating and cell culture as described above. As for the Brick Strex S device, we provide a complete list of parts and assembly instructions and videos for the Brick Strex L (Supplementary Table 2, Supplementary Fig. 2–4, Supplementary Movie 2).

Accurate measurement of the membrane strain is highly important in the stretching and compression experiments. This was achieved in Brick Strex S and L devices by using a caliper to measure the membrane position before and after manipulating the membrane strain. In addition, we designed a simple add-on module with a printable scale to the devices, which allows continuous measurement of the device strain during the experiment (Supplementary Figs. 5 and 6). Due to the minimalistic and simple design, both devices are low-cost, cost-effective, and easy to use without any special expertise or maintenance. We have summarized their key features in Supplementary Table 3 for quick comparison of our devices with other similar systems.

### Intracellular localization of $\upbeta$-catenin and YAP in response to increased lateral stretching or compression with Brick Strex S

After constructing the device, we carried out lateral stretching and compression experiments with MDCK type II epithelial cells and Brick Strex S. Here, we analyzed for the cellular mechanical responses by determining the intracellular localization of $\upbeta$-catenin and/or YAP1. The Wnt/$\upbeta$-catenin pathway and subcellular localization of $\upbeta$-catenin and YAP1 are known to be affected by the cell density, extracellular environment stiffness and modulation of the intercellular junctions^42–44^. In a confluent epithelium, $\upbeta$-catenin localizes to the adherence junctions at the plasma membrane via association to E-cadherin^23,43,45^. In addition, when the Wnt pathway is turned off, β-catenin is constantly being recruited to the “destruction complex” and degraded by proteasomal degradation preventing β-catenin nuclear targeting^46,47^. Activation of the Wnt pathway results in inhibition of β-catenin breakdown allowing its accumulation, nucleus entry, and finally β-catenin-mediated activation of Wnt target genes^46,47^. Moreover, mechanical stretching of confluent epithelium has also been shown to lead into nuclear accumulation of β-catenin^23^. Respectively, a dense growth condition increases Hippo pathway regulator YAP1 phosphorylation especially from serine 127 (S127) driving YAP1 sequestration, proteasomal degradation and/or retention in the cytoplasm^23,48^. In contrast, in sparse growth density, loss of contact inhibition and/or promotion of cellular proliferation, YAP1 is mostly unphosphorylated and predominantly located in the nucleus^48–50^. In addition, increased mechanical tensile strain has been shown to drive YAP1 nuclear localization^28,48^.

**Figure 2:**
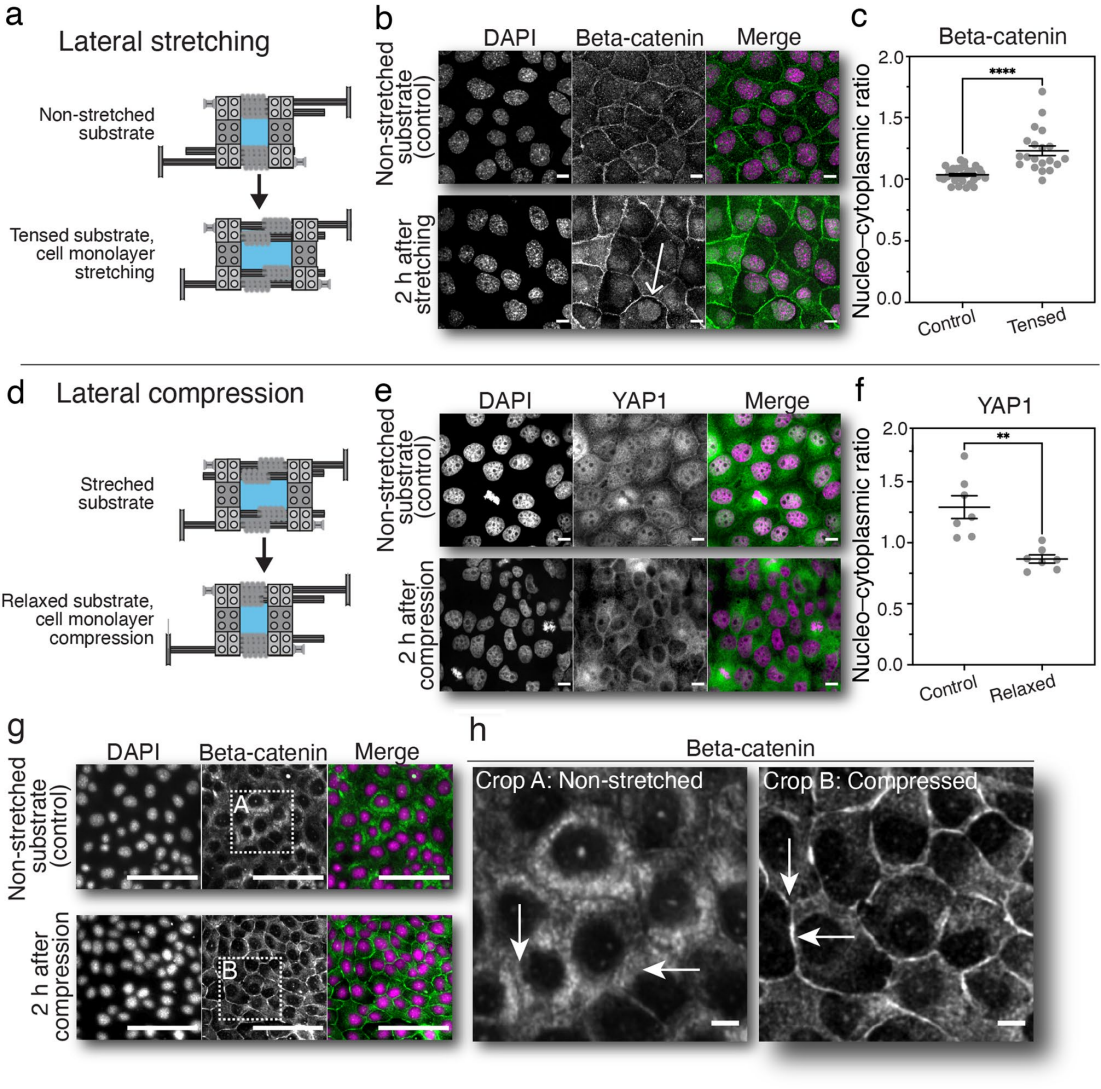
Manual cell stretching and compression experiment with Brick Strex S. Epithelial cell monolayers were grown on silicon membranes and subjected to stretching or compression in Brick Strex S device. (**a**) Epithelial stretching experiment, where epithelium was subjected to a 25% strain. (**b**) Representative maximum intensity Z-projection confocal microscopy images showing cells before (relaxed substrate) and after stretching (2 h after stretching). Cell were stained with DAPI to highlight the nuclei (magenta) and immunolabelled against $\upbeta$-catenin (green). (**c**) Quantification of the nucleo-cytoplasmic ratio of $\upbeta$-catenin after single stretch of the substrate in comparison to unstretched control samples. (**d**) Lateral compression experiment of the epithelial monolayer. (**e**) Cells were stained with DAPI to highlight the nuclei (magenta) and immunolabelled against YAP1 (green). (**f**) Quantification of the nucleo-cytoplasmic ratio of YAP1 in response to compression. (**g**) Similar lateral compression experiments as shown in e. The cells were stained with DAPI to highlight the nuclei (magenta) and immunolabeled against $\upbeta$-catenin (green). (**h**) blow-up images of the $\upbeta$-catenin staining shown in g. Scale bars 10 $\upmu$m. Error bars represent the standard error of the mean (SEM). Statistical analyses were performed using an un-paired Student´s t-test when comparing between cells before and after stretching/compression (****p < 0.0001; **p < 0.05).

In our stretching experiments, PDMS membrane clamped to the Brick Strex S was first coated with fibronectin. Next, the cells (2 × 10^4^ cells/1.1 cm^2^) were seeded on top (Fig. 2a). On the following day, the epithelium was stretched to a 25% tensile strain by manually turning the device axles for ¼ of a turn (equal to approx. + 6.25% increase in tensile strain) four times with 10-min intervals. The cells were let to respond to the substrate stretching for 2 h within the incubator followed by fixing. To investigate the localization of $\upbeta$-catenin in the stretched epithelium, we stained our samples against $\upbeta$-catenin and F-actin (phalloidin) (n = 2). We found that in the control epithelium cultured on a non-stretched membrane, $\upbeta$-catenin was predominantly cytoplasmic with a slight accumulation to the cell vertexes. In contrast, stretching with the Brick Strex S induced increased nuclear localization of $\upbeta$-catenin (Fig. 2b), which was quantified by measuring the nucleus-to-cytoplasm ratio of $\upbeta$-catenin (Fig. 2c). This result is in line with the previous reports of $\upbeta$-catenin nuclear localization after single stretch of epithelium^33^.

Next, we wanted to study the effects of uniaxial lateral compression (Fig. 2d) on the mechanotransduction signaling within the epithelium. This was achieved by seeding 2 × 10^4^ cells into a cell culture column attached to a fibronectin coated PDMS membrane stretched into a 25% tensile strain on Brick Strex S with double axles. Concurrently, cells were equally grown on a non-stretched membrane to achieve non-compressed control sample. Cells were grown until a desired confluency (approx. 100%) was reached and the cells were in physical contact with each other. The compression experiment was done sequentially by turning the axles ¼ of a turn (equal to approx. −5% compressive strain) in 10-min intervals until full relaxation was achieved. The following compressive strain was −20%, due to the relaxation from prior 25% tensile straining of the substrate (Eq. 1, $\varepsilon = \frac{1 - 1.25}{{1.25}} = - 0.2)$. Cells were kept in the incubator during the recovery between the relaxation sequences. Cells survived the careful and slow manipulation of the membrane and remained attached to the surface. No significant cell death was observed. Finally, the compressed cells were fixed at 2 h post substrate manipulation together with control samples and immunostained against YAP1 (Fig. 2e) or $\upbeta$-catenin (Fig. 2g). We speculated that as opposite to stretching, the lateral compression would lead into an increase in cell density and growth-inhibitory mechanical signaling. In the non-compressed less dense control epithelium, YAP1 was mostly nuclear, while in the compressed epithelium with a forced higher cell density YAP1 was mainly cytoplasmic (Fig. 2f). Of note, immunolabeling of the cells grown on the stretched and unstretched membranes lead into the development of identical epithelial monolayers as detected by the nucleus, actin and YAP1 staining, and by quantification of the number of cells per field of view and YAP nucleo-cytoplasmic ratio (Supplemental Fig. 7, n = 2). These indicate that the 25% tensile strain in the membrane used in the experiments does not influence the epithelial growth, maturation, or appearance. Like YAP1, also $\upbeta$-catenin showed sensitivity to lateral compression of the monolayer. Specifically, in the non-compressed epithelium, $\upbeta$-catenin was mainly located in the cytoplasm and to some extent on the plasma membrane. We did not detect $\upbeta$-catenin in the nucleus. In contrast, in the compressed monolayer $\upbeta$-catenin was less pronounced in the cytoplasm but significantly accumulated into the cell–cell junctions (Fig. 2g,h, n = 2). Together, these results show that increased intercellular compression leads into cytoplasmic retention of YAP1 and decreased cytoplasmic presence and concerted relocalization of $\upbeta$-catenin to the plasma membrane.

### Biochemical analyses of YAP1 and $\upbeta$-catenin under increased intercellular compression with Brick Strex L

Immunostainings of the compressed epithelium suggested that YAP1 becomes cytoplasmic and that $\upbeta$-catenin becomes less pronounced within the cytoplasm relocalizing to the cell–cell contacts in response to increased lateral compression. It is acknowledged that in full confluency and in the absence of growth-promoting signaling such as Hippo and Wnt, YAP1 is phosphorylated from serine 127 (S127) enhancing its cytoplasmic retention and degradation^49^. In concert, the cytoplasmic pool of $\upbeta$-catenin is constantly cleaved and degraded^46^. Via these aforementioned mechanisms, both YAP1 and $\upbeta$-catenin are excluded from the nucleus preventing their role as a transcriptional activator. The membrane-association of $\upbeta$-catenin with E-cadherin has been suggested to require inhibition of proteolytic cleavage leading to its stabilization and accumulation of heavy multi-ubiquitinated forms^47,51^. We postulated whether the compressive mechanical stress could enforce negative signaling of growth and proliferation. To test our hypothesis and to detect for the phosphorylation of YAP1 and the cytoplasmic presence of YAP1 and $\upbeta$-catenin, biochemical analysis was required. To this end, the cells were grown on both non-stretched and stretched (25% tensile strain) fibronectin coated PDMS membranes on the Brick Strex L (n = 2) enabling larger amounts of cells to be grown for the biochemical analyses (n = 2). After two days of growth and acquiring of confluent growth conditions, the relaxation of the membrane i.e., compression of the cells was accomplished (compressive strain −20%). After a 2 h recovery, the cells were washed and lysed for western blot.

**Figure 3:**
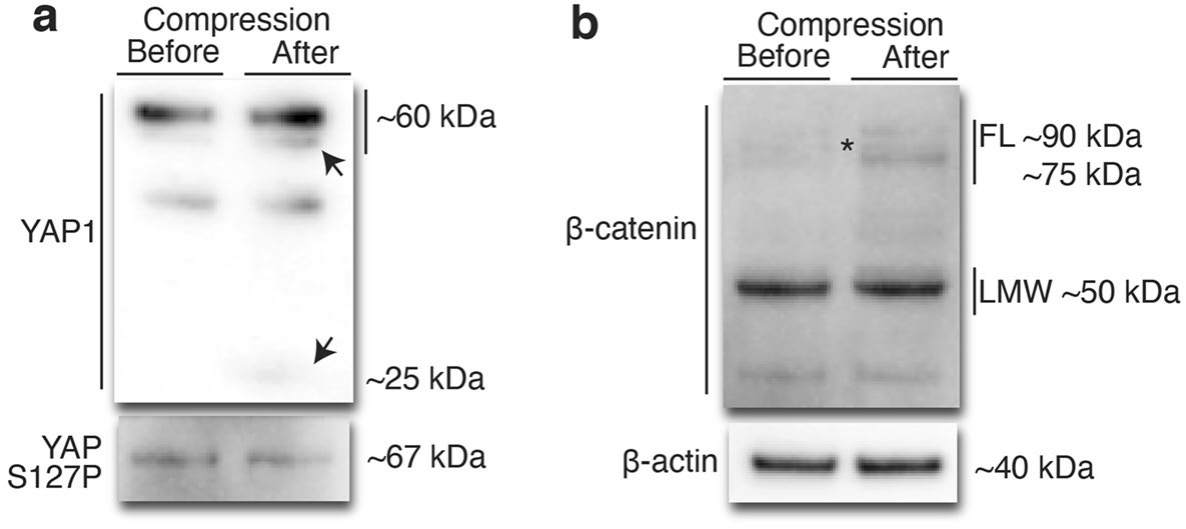
Western blot analysis of lateral compression -induced effects on YAP1 and $\upbeta$-catenin. Epithelial cells were cultured on a pre-strained PDMS membrane in Brick Strex S device until full confluency. Following sequential membrane relaxation and increased lateral compression, the cells were let to recover for 2 h prior to cell lysis and analyzed by western blot (n = 2). (**a**) Expression and phosphorylation (S127P) status of YAP1 and (**b**) expression of $\upbeta$-catenin were analyzed from whole cell lysates of non-compressed (before) and compressed (after) samples (20 $\upmu$g of total protein per sample). Arrows indicate the compression-induced cleavage products of YAP1. Asterisk indicates the presence of full-length (FL) and higher molecular weight $\upbeta$-catenin in the compressed sample. $\upbeta$-actin was used as an internal control. See also full-length blots in Supplementary Fig. 8.

First, the expression and phosphorylation status of the transcription regulator YAP1 of the Hippo-pathway were studied. The analysis showed that in comparison to the non-compressed control, compression led to the presence of low molecular weight cleavage products of YAP1 suggesting for its proteolytic degradation or cleavage (Fig. 3a; see also full-length blots in Supplemental Fig. 8). In addition, YAP1 S127 phosphorylation levels remained nearly unaltered after the compression in comparison to that in the non-compressed confluent control epithelium (Fig. 3a, lower panel; see also full-length blots in Supplemental Fig. 8). Together, these results suggest for enforced retention and degradation of YAP1 in the cytoplasm in response to increased lateral compression.

Next, the expression and mobility shift of $\upbeta$-catenin were determined. The experiment showed that in the non-compressed confluent epithelium, the concentration of full length $\upbeta$-catenin was low while low molecular weight fraction was apparently indicative of $\upbeta$-catenin degradation within the cytoplasm. In contrast, the compression led to an increase in the presence of the full-length $\upbeta$-catenin and in the emergence of higher molecular weight forms. This suggests for increased accumulation of post-translationally modified forms of $\upbeta$-catenin, inhibition of proteolytic degradation, and stabilization of the protein referring to inhibitory growth signaling. The results are in line with earlier studies showing that membrane-association of $\upbeta$-catenin is preceded by increased post-translational modification and inhibition of its cleavage^47^. However, the low molecular weight fraction was detected both in compressed and non-compressed control samples (Fig. 3b; see also full-length blots in Supplemental Fig. 8). Our immunostaining experiments (Fig. 2g) together with the western blot studies enforce the view that the lateral mechanical compression induces localization of the $\upbeta$-catenin at the cell–cell junctions and inhibition of $\upbeta$-catenin -mediated growth signaling.

### Live-cell imaging of lateral compression -induced cellular packing with Brick Strex L

**Figure 4:**
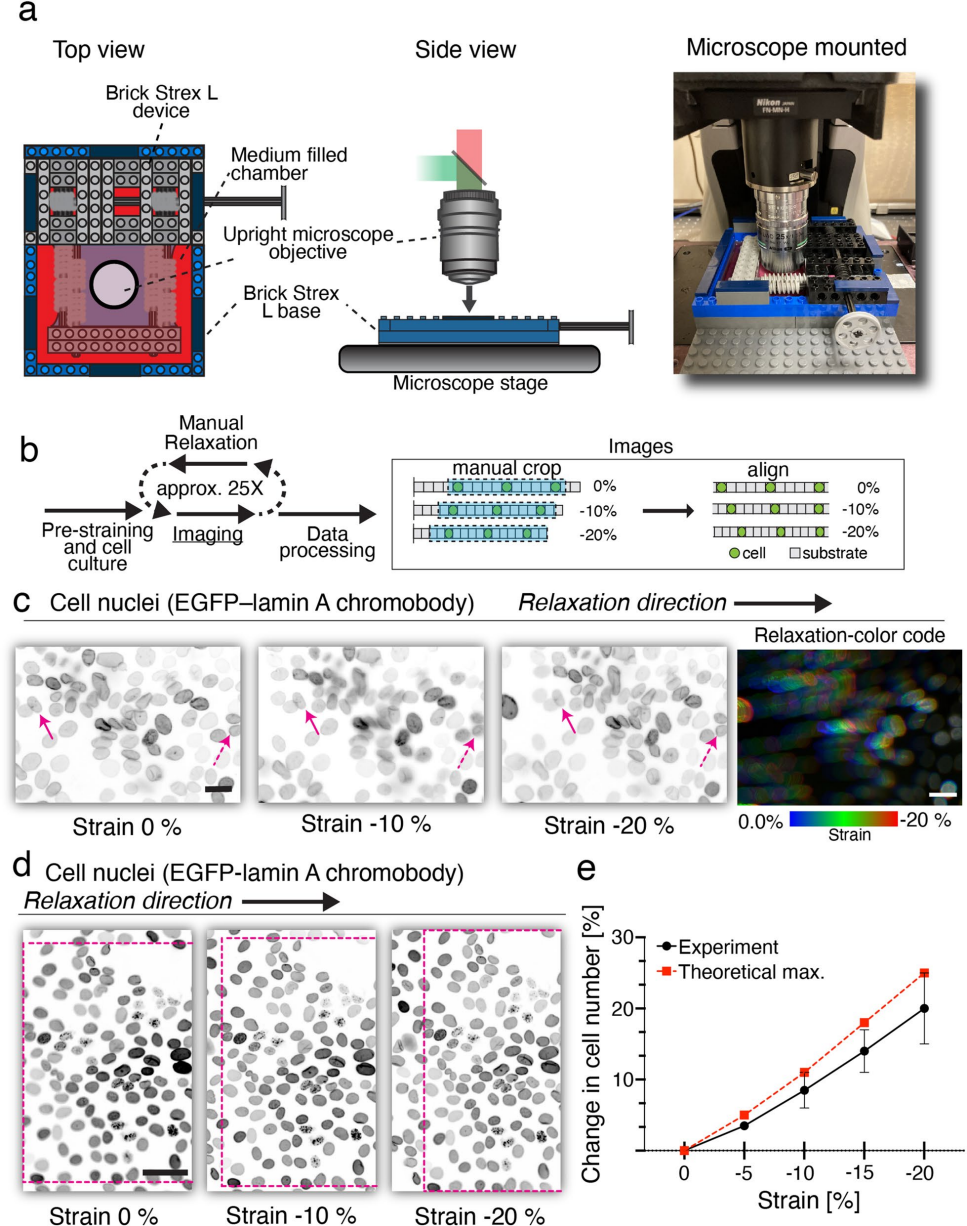
Manual live-cell compression studies with Brick Strex L. Monolayers of epithelial MDCK II cells stably expressing EGFP – lamin A chromobody were grown on stretched PDMS membranes and subjected to manual lateral compression in Brick Strex L device mounted on inverted microscope. (**a**) Schematic image of the experimental setup used in the manual relaxation (left and middle panels). Device L mounted on an upright microscopy stage (right panel). (**b**) Scheme for the manual relaxation experiment. The membrane was subjected to 25 relaxation—imaging cycles. Data was processed by cropping and aligning the images. (**c**) Microscopy images showing packing of the nuclei during the subsequent manual relaxation steps. Arrows indicate the location of the same nucleus during the relaxation, dashed arrows show the nucleus used in the data alignment. Scale bar 20 µm. (**d**) Bigger field of view of manual stepwise relaxation (approx. 1% strain relaxation steps) experiment and packing of cells. Rectangular area highlights the epithelial deformation. Scale bar 50 µm. (**e**) Experimentally derived (black curve) and calculated theoretical maximal (red curve) percentage change (%) in the number of cells per field of view as a function of the compressive strain. Error bars represent the standard error of the mean (SEM).

Brick Strex S and Brick Strex L allowed mechanotransduction studies of adherent cells by using immunofluorescence labeling and western blot. Following these experiments, we wanted to investigate the possibility to use Brick Strex L in live cell imaging. Live cell imaging studies offer a possibility to follow cellular dynamics during or immediately after cellular manipulation and thus are a vital part of mechanobiological readouts. In line with this, the Brick Strex L device allows the imaging of the cells by using upright epifluorescence microscopy (widefield or confocal) (Fig. 4a). For the initial studies we used MDCK cell line stably expressing EGFP—lamin A chromobody, a nuclear lamina marker. The cells were seeded on 25% -stretched membrane on Brick Strex L and cultured until confluency (n = 2). The device was mounted on the sample stage and the tensile 25% stretch was manually relaxed in approximately −1% increments and imaged in between the relaxation steps (Fig. 4b), leading into −20% compressive strain. Before the image capture, the stage was moved and refocused, allowing the imaging of the same field of view throughout the relaxation. The imaging data was then cropped and aligned (Fig. 4b). The analysis showed that the relaxation led to lateral compression and increased nuclear packing of the EGFP – lamin A chromobody -expressing cells (Fig. 4c).

To analyze further the cellular packing in compression, MDCK cells expressing the fluorescent nuclear lamin chromobody were again used together with Brick Strex L and cultured until confluency (n = 2). The compression was done by subsequent manual relaxation as described above and the same sample position was imaged by moving the microscope stage to compensate the lateral movement of epithelium during the compression. Similarly as before, the compressive strain was −20%, due to the relaxation from the 25% tensile strain of the substrate (Eq. 1, $\varepsilon = \frac{1 - 1.25}{{1.25}} = - 0.2)$. The collected data was manually aligned, and the cell numbers were calculated from the fields (Fig. 4d). The −20% compressive strain lead into a 20% increase in the cell density (Fig. 4e). Theoretically we can calculate (according to the Eq. 4), that upon a −20% lateral compressive strain of the material with a Poisson ratio of 0, the cell density should increase by 25% (Supplementary Fig. 9). Nevertheless, this indicates that Brick Strex can be used with living cells to follow changes in cellular organization.

Together, these experiments show that the manual relaxation of the substrate leads to compression of the epithelium leading to a significant increase in the higher monolayer packing.

### Live cell imaging using motorized Brick Strex L

**Figure 5:**
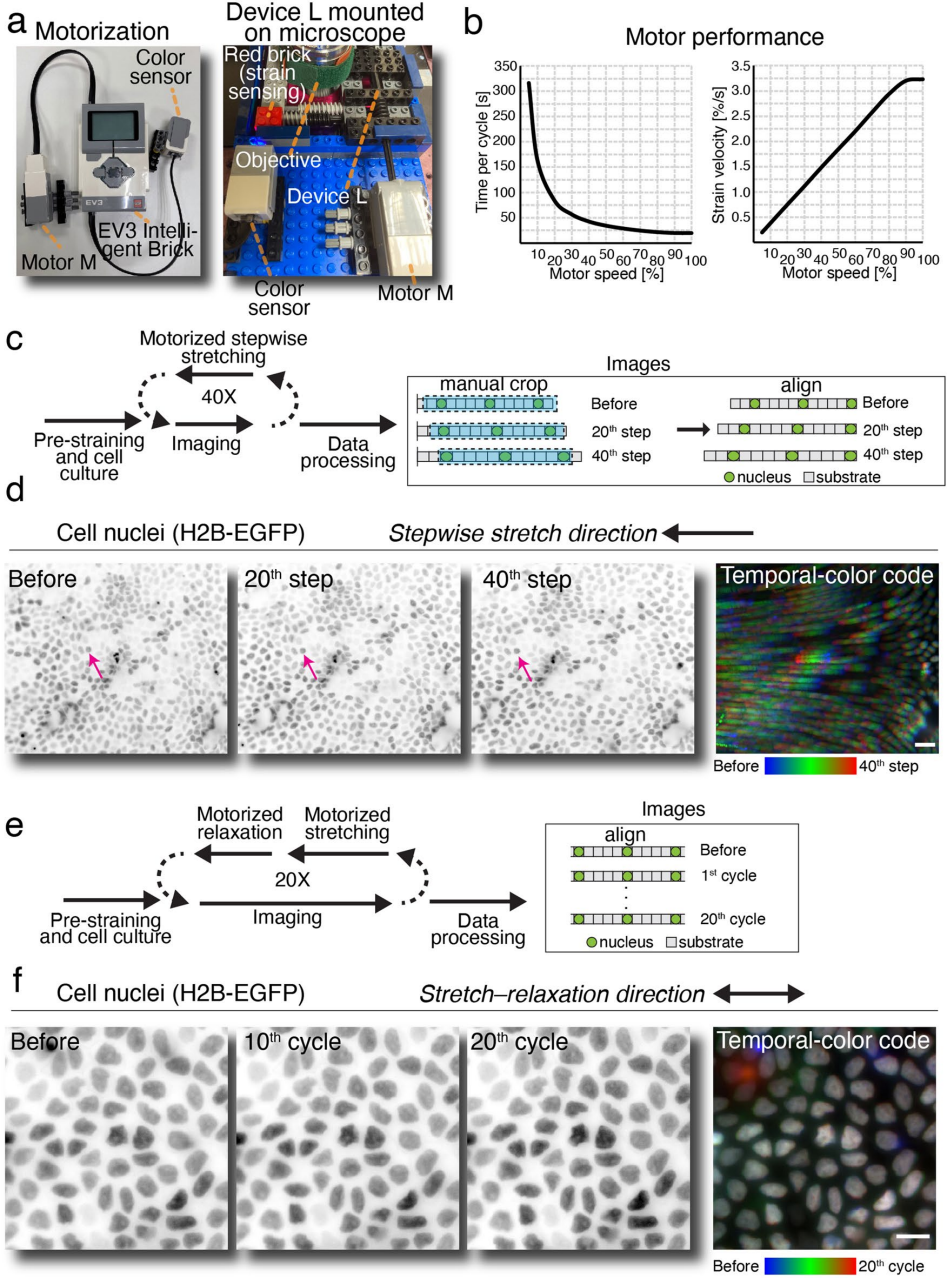
Automated live-cell compression studies with Brick Strex L. Motorized manipulation of MDCKII cells stably expressing histone H2B-EGFP was done by using (**a**) Brick Strex L device connected to a LEGO EV3 Intelligent Brick, motor M and color sensor (left panel; see also Supplementary Fig. 10), followed by mounting on an upright microscope stage (middle panel). (**b**) Motor performance was quantified by measuring the time for one full cycle of movement with different motor speeds. The strain velocity, measured as strain-% / s, is highly linear function of the motor speed setting. **c**, Scheme for the automated stepwise relaxation experiment. (**d**) Cells were grown on stretched membrane, after which the membrane was subjected to 40 relaxation steps, imaged between the steps and the data was processed by aligning the images. Arrows indicate the location of the same nucleus during stretching. Scale bar 50 $\upmu$m. (**e**) Scheme for the automated cyclic relaxation-stretching experiment. (**f**) Relaxation-stretching cycles performed for 20 times. Temporal-color code presenting the minimal nuclei movement in response to the lateral compression. Scale bar 25 $\upmu$m.

To further automate the cell stretching and compression experiment, we combined Brick Strex L and commercially available LEGO EV3 Intelligent Brick and motor (M size) (Fig. 5a, right panel). The motors and the whole manipulation processes is then easily programmable by using the LEGO Mindstorms software. This allowed automation and remote control of the stretching experiments and ensured even cyclic manipulation. First, we characterized the Brick Strex L performance with the motor. The maximal tensile strain of the system was 31.4%. At the full speed of the motor (speed 100/100), the stretch-relaxation cycle took 19.5 s which corresponds to 3.2% tensile strain/s velocity and at lowest motor speed setting of 5/100, the cycle took 316.1 s, corresponding to 0.2% tensile strain/s velocity (Fig. 5b).

Next, MDCK cells stably expressing histone H2B-EGFP enabling visualization of the nuclei in real time during the manipulation were seeded as described above onto a PDMS membrane. After growing until a full confluency, the monolayer was stretched in stepwise manner into a 25% tensile strain with 40 subsequent motor-based axle rotations. Imaging of the same field of view was done after each stretching step to observe the effects on the cells (Fig. 5c,d). The data shows clear stretching of the epithelial monolayer as indicated by the movement of the cell nuclei.

To achieve cyclic stretching possibility, a built-in rotation sensor of the motor and additional color sensor of the EV3 system were used with the Brick Strex L device (Fig. 4a). This allowed an easily programmable cyclic manipulation and driving the manipulation with a mobile phone, tablet, or a laptop (Supplementary Fig. 10 and 11). The color sensor was used to define the endpoint of the stretching and built-in rotation counter was then used to achieve constant relaxation of the device (Fig. 5a). The setup allowed the imaging of the same position in the sample and showed surprisingly high reproducibility in the movement of stretching of the membrane. When stretching was conducted for 20 cycles with a 25% maximal tensile strain, the average drift of the field was 160 ± 85 µm per one cycle corresponding to a 0.5% difference in the tensile strain. Next, we cultured MDCK cells expressing histone H2B-EGFP on a PDMS membrane in the Brick Strex L and studied the effects of cyclic motorized stretch-relaxation cycles on the epithelium. To this end, the motor system allowed a robust control of the experiment: we performed twenty (20) stretch–relaxation cycles and imaged the same field between the cycles (Fig. 5e, n = 2). The imaging was conducted with an upright fluorescence microscope. The imaging focal plane remained surprisingly stable throughout the experiment. However, the cyclic manipulation had only a minor effect on the closely packed epithelial nuclei (Fig. 5f). The nuclei did not show any morphological changes, and orientation and localization did not change during the stretching cycles.

Taken together, here developed Brick Strex S and Brick Strex L devices allow versatile and robust cell stretching or compression experiments. Our proof-of-principle experiments show that the manipulation can be easily combined with immunofluorescence imaging, biochemical readouts, or even live-cell microscopy. By using the devices, we were able to show how stretching or lateral compression of epithelial monolayers influence $\upbeta$-catenin and YAP1 -related mechanosignaling.

## Discussion

Tissues encounter both intrinsic and extrinsic mechanical forces that can influence cellular functions and behavior^52^. The force-related cellular responses have remained understudied partially due to the lack of proper tools allowing easy mechanical manipulation of cells and tissues. Furthermore, challenges emerge also from the complexity of the cell and tissue viscoelastic properties, and the magnitude, loading rates and timescales of experienced strains^44^. For example, alveolar epithelium of the lungs can experience over 25% tensile strains and the small intestine mucosa even 40% continuous compressive strain^53,54^. Additional level of complexity rises from the possible collective behavior of the cells e.g., in the epithelium^55^. Finally, during the recent years it has become evident that in addition to cell membrane associated adhesion sites, forces are sensed deeper within the cells^16^ Mechanical forces rising from the ECM can be transmitted into the cells and they have been shown to directly affect even cell nuclei and chromatin^56,57^. Therefore, the tools used in the mechanobiological research should facilitate the use of advanced microscopy methods, biochemical assays, next-generation sequencing and other -omics tools, allowing direct investigation of the force induced effects in signaling pathways, chromatin and other nuclear structures.

In the last two decades, various approaches have been developed to study especially the effect of mechanical stretching on cells and tissues. This has been achieved by constructing either mechanically or pneumatically actuating devices^58^. In addition, few commercial solutions are also available (e.g., Flexell, CellScale). These approaches offer precise and highly reproducible ways to conduct cell stretching experiments but often require deep expertise in engineering and manufacturing processes, not necessarily available in every institute. More recently, Boulter et al. constructed a motorized stretching device from homemade elastic silicon elastomer vessel and LEGO blocks, lowering the manufacturing threshold of such devices^59^. In line with this, we focused on developing a simple, robust, reproducible, well controllable, easily approachable and reconfigurable platform to study how uniaxial stretching or compression affects cells. The Brick Strex devices presented here are easy to assemble, and we provide detailed instructions on how they are constructed. They can be operated manually, or the larger device can also be motorized by the LEGO EV3 motors and controllers. In addition to the bricks, the system needs only commercially available silicon membrane, thus additional construction steps or parts are not necessary. Thus, they provide a low threshold approach for mechanical manipulation of cells, and we envision that they can be easily used also for educational purposes. By using the Brick Strex devices, we show that increased uniaxial inter-cellular i.e., lateral compression results in modulation of YAP1 and $\upbeta$-catenin signaling.

To prove the suitability of the Brick Strex in mechanobiological studies, we first applied lateral stretching to the cells and detected for intracellular localization of $\upbeta$-catenin. In the epithelium, $\upbeta$-catenin is located either in the intercellular junctions as plasma membrane -bound, as cytoplasmic and/or as nucleoplasmic fractions^7^. Once membrane-bound, $\upbeta$-catenin has important adhesion functions, whereas in the nucleus it serves in activation of transcription in the Wnt growth factor signaling pathway^60,61^. Stretching of epithelium has been shown to lead into release of $\upbeta$-catenin from E-cadherin and its nuclear accumulation, which has been visible even 16 h post stretching^23,60^. This was also evident in our stretching experiment with Brick Strex S and immunostaining of $\upbeta$-catenin. Thus, these experiments indicate that the concept can be used in mechanobiological research, and it provides similar cellular responses as previously published devices.

Next, our main aim was to analyze the mechanical responses to increased lateral forces and performed lateral compressions with the Brick Strex device. For this, we detected for the intracellular localization of YAP1 and $\upbeta$-catenin—known mechanosensitive co-regulators of cellular plasticity. Here, our Brick Strex -induced compression experiments pointed to the direction of negative growth signaling: our immunostainings showed that the lateral compression led to uniaxial contraction of the cell monolayer that induced high cell density, cytoplasmic retention of YAP1 and triggered relocalization of $\upbeta$-catenin to the cell membrane. The acquired data from the biochemical analyses further indicated that compression induced cleavage of YAP1 but did not affect YAP1 phosphorylation at S127P. Cytoplasmic retention of YAP is associated with the suppression of its nuclear activity and increased contact inhibition and Hippo signaling^49^. Here, the compression promoted the expression of full-length $\upbeta$-catenin together with higher molecular weight fractions suggestive of β-catenin membrane-association and inhibition of β-catenin -mediated growth signaling. Indeed, earlier studies have reported the presence of high molecular weight bands in the membrane-containing cell lysate fractions^47^. Moreover, the high molecular weight β-catenin have been shown to be associated with increased post-translational modifications and suggested to be a prerequisite for E-cadherin tethering and inhibited proteolytic cleavage^47^. Interestingly, the Hippo pathway-dependent S127 phosphorylated form of YAP1 has been shown to participate in retaining $\upbeta$-catenin in the cytoplasm and to suppress its signaling through direct binding^62^. In addition, inhibition of Wnt/$\upbeta$-catenin signaling by ubiquitinylation has been shown to be YAP-dependent in cancer^63^. Along these lines, our western blot analyses suggest for increased $\upbeta$-catenin membrane-association and/or suppression of beta-catenin signaling in response to lateral compression. Recent advances on cell–cell junction mechanics have indicated that mechanical forces sensed at the junctions lead to distinct mechanical responses at the junctions^29^. Thus, it can be hypothesized that the compression-induced membrane tethering of β-catenin is related in enforcement of the intercellular junctions upon increased mechanical loading. To conclude, by using the Brick Strex mechanical manipulation devices we were able to show that increased lateral compressive forces induce cytoplasmic retention of phosphorylated YAP1. Importantly, we report for the first time that increased uniaxial lateral compression leads to increased membrane-association of β-catenin promoting contact inhibition driving negative regulation of the growth and proliferation of the confluent epithelium.

Live cell imaging is a widely used methodology in biomechanics and mechanobiology research. Importantly, Brick Strex L also allows fluorescence microscopy of the manipulated living cells by using an upright microscope. We demonstrated this by conducting manual compression experiments, where fluorescent nuclear lamina binding chromobodies were used to highlight the cell nuclei in the confluent epithelium. The lateral compression lead into rapid packing of the cell monolayer as quantified by measuring the increase in the number of cells in the field of view as a function of the compressive strain. The difference between the theoretical maximal and the experimentally determined increases in the cell number can be explained by local variation in the cell growth density on the membrane and non-zero Poisson ratio of the membrane.

Finally, we motorized the device and applied it in live cell microscopy to detect dynamic mechanical changes in the epithelial monolayer. To this end, cells grown to equal confluences on pre-stretched membranes were exposed either to single stepwise stretching or cycles of stretching-relaxation manipulation. The automation of the cyclic process accomplished by connecting the device to LEGO EV3 Intelligent Brick motor and LEGO Mindstorms Home software. The results showed that single stepwise stretching of the substrate lead to corresponding stretching of the epithelial cell layer. However, the cyclic uniaxial stretching-relaxation of the epithelial cells affected neither the morphology nor the orientation of the nuclei. In earlier reports, cyclic mechanical manipulations have led to a tensile strain avoidance response, where myofibroblast cells reorient and change their position away from the direction of the applied strain^64^. The orientation of fibroblast cell nuclei has also been shown to respond to equibiaxial cyclic stretching of the substrate^56^. Along these lines, our experiments might indicate that the nuclear responses to mechanical stress could be dependent on the straining parameters (loading rate, duration, etc.) or the studied cell types.

To conclude, the Brick Strex devices are well suited to the biochemical analyses of the cells. With these easy and robust devices, we studied the effects of mechanical force on cells using lateral uniaxial lateral compression and immunohistochemical analyses. The experiments presented here prove that the device can be used to trigger mechanotransduction in the epithelium promoting reproducibility and consistency in quantitative mechanobiological studies without costly equipment. As a result, we obtained both fixed and live cell imaging microscopy data on the compressed cells, and finally biochemical analysis data on the modulation of the mechanotransduction signaling without special technical expertise. The application of cost-effective in-house built devices to study epithelial force transduction has a great application value from the methodology perspective. With this being said, the Brick Strex devices with larger sample sizes allow also genetic analyses of mechanically stretched or compressed cells. In addition, the devices have a wide reach and potential utilization value in the scientific community including teaching and related pedagogical actions. All in all, the presented mechanical manipulation devices with the aforementioned methodological improvements will enable us to better understand important processes of mechanotransduction and their effect on cellular physiology.

## Methods

### Cell culture

Madin-Darby canine kidney (MDCK) type II, and MDCKII cells stably expressing EGFP – lamin A chromobody or histone H2B-EGFP were maintained in Modified Eagle´s medium (#51,200,046, Thermo Fisher Scientific, Waltham, MA, USA) supplemented with 1% (vol/vol) penicillin–streptomycin antibiotics (#15,140,122, Thermo Fisher Scientific) and 10% fetal bovine serum (#10,500,064, Thermo Fisher Scientific) under standard conditions in a humidified cell incubator (+ 37 °C, 5% CO_2_). For the lateral compression experiments, 2 × 10^4^ cells for microscopy imaging (Brick Strex S) or 8 × 10^4^ cells for biochemical assays and live cell studies (Brick Strex L) were seeded in cell culture columns attached onto fibronectin (10 µg/mL) -coated and UV-treated (30 min) thin (0.01") USP class VI PDMS membranes (8 × 4.5 cm, Specialty Manufacturing, Inc., Saginaw, MI, USA) using high-vacuum silicone grease (Dow Corning, Merck, Darmstadt, Germany). The PDMS membranes were assembled in Brick Strex with 25% strain (measured as 25% increase in stretch). The columns were prepared by cutting the column off from a PET insert (small S device) or by using 8.8 cm^2^ plastic ring with a height of 1 cm (Brick Strex L). As a control, cells were seeded also on unstretched PDMS membranes in similar columns. Cells were grown until a desired confluency was reached.

### Device construction and characterization

The Brick Strex devices (Fig. 1) were assembled of LEGO bricks (Supplementary Table 1 and 2, and supplementary Figs. 1–4) and detailed assembly instructions are provided in the supplements (Supplementary Movie 1 and 2). Strain was calculated according to

1 $$\varepsilon = \frac{{l - l_{0} }}{{l_{0} }}$$

where ε is strain, l is the resulting length of the object and l_0_ is the original length of the object. A 25% tensile strain ($\varepsilon = \frac{1.25 - 1.0}{{1.0}} = 0.25)$ was applied in all the experiments leading into a 20% compressive strain in the lateral compression experiments where the membrane was relaxed ($\varepsilon = \frac{1.0 - 1.25}{{1.25}} = - 0.2$). The theoretical change in the cell density d due to the compression was calculated accordingly

2 $$\frac{density\,after}{{density\,before}} = \frac{{d_{after} }}{{d_{before} }} = \frac{{\frac{n}{{A_{after} }}}}{{\frac{n}{{A_{before} }}}} = \frac{{A_{before} }}{{A_{after} }}$$

where n is the number of cells (assumed to stay constant during the experiment) and A is the rectangular cell substrate area before and after the compression. In the case of uniaxial stretching or compression (strain > −1) of a rectangular area and when assuming that material Poisson ratio is 0 (deformation only along the x-axis) we get

3 $$A_{after} = x_{after } * y_{after } = \left( {1 + \varepsilon } \right)*x_{before } * y_{before } = \left( {1 + \varepsilon } \right)*A_{before}$$

where ε is the strain, and x_after_, y_after_ are x and y dimensions of the area after the strain and x_before_, y_before_ are x and y dimensions of the area before the strain. By placing Eq. (3) into the Eq. (2) we get,

4 $$\frac{density\,after}{{density\,before}} = \frac{{d_{after} }}{{d_{before} }} = \frac{{A_{before} }}{{\left( {1 + \varepsilon } \right)*A_{before} }} = \frac{1}{{\left( {1 + \varepsilon } \right)}}$$

In the Eq. (4) we assume that the membrane Poisson ratio is 0. With PDMS membranes this is not the case and therefore the Eq. (4) gives the maximal increase in the cell density during compressive strain.

The strain was measured by using a caliper before and after membrane stretching/relaxation or by using additionally an add-on module with a scale (Supplementary Figs. 5 and 6). Brick Strex L device base was sealed to avoid medium leaks with poly(dimethylsiloxane) (PDMS) (1:10 w/w, curing agent: Sylgard 184 pre-polymer, Merck). For motorization of Brick Strex L device, LEGO servomotor M was used together with color sensor (Supplementary Fig. 10). The motor was controlled with a LEGO EV3 Intelligent Brick. Cyclic and/or stepwise stretching/compression experiments were programmed using LEGO Mindstorms software (version 1.5.0, available at https://www.lego.com/en-us/themes/mindstorms/downloads) (Supplementary Fig. 10 and Supplementary Movie 3). The motor was operated at full speed, corresponding 3.2% strain / s velocity. The reproducibility of the stretching cycles was measured by following the position of single cell within the same field of view during the 20 cycles of stretching.

For plating the cells, Si membrane was clamped to the device, a cell culture column was attached using silicone grease, and the membrane was coated with fibronectin and treated with UV for 30 min. Next, the excess fibronectin was aspirated, and the membrane was washed once with PBS. Medium (2 mL) was added to the column and the whole device was brought into the cell incubator 15 min prior plating the cells for stabilization of the pH and temperature. To characterize the uniaxial stretch, a 3D printed mold was used to mark the stretched PDMS membrane with an array of dots, or by applying fluorescent beads on the stretched membrane (FluoSpheres Polystyrene Microspheres, 1.0 $\upmu$m, yellow/green fluorescent (505/515), Thermo Fisher Scientific) (data not shown). The membrane was then imaged with Nikon AZ100 Multizoom upright fluorescence macroscope with built-in 8X zoom optics using a 5X objective (Nikon AZ-Plan Fluor 5x/0.50, working distance (WD) 15.0 mm, air). Excitation was done by using C-HGFI Precentered Fiber epifluorescence 100 W mercury illuminator as a light source and emission was collected by using 520/35 filter. Image capturing was done with DFK 33UX250 CMOS color camera. The quality of stretch was analyzed by measuring the distance of the drawn points prior and post the relaxation of the membrane with a digital caliper (data not shown).

### Immunolabelling

Immunostaining of the samples was done within the attached cell culture columns by using a primary rabbit antibody against $\upbeta$-catenin (1:2000 in 3% BSA-PBS, ab6302, Abcam, Cambridge, UK), a mouse monoclonal antibody against YAP1 (1:500 in 3% BSA-PBS, YAP163.7, sc-101199, Santa Cruz Biotechnology, Dallas, USA) and Alexa 568-conjugated phalloidin to detect actin (1:100 in 3% BSA-PBS, 1 h in RT, in dark) followed by Alexa 488-conjugated goat anti-mouse and Alexa 647-conjugated goat anti-rabbit secondary antibodies (Thermo Fisher Scientific, Waltham, MA, USA). The entire immunostaining method is described in more detail in Supplementary methods online.

### Microscopy

Confocal microscopy of the fixed samples was conducted using Nikon A1R laser scanning confocal microscope mounted in inverted Nikon Ti–E (Nikon Instruments Europe BV, Amsterdam, Netherlands). The excitation laser lines and emission filters were 405 nm and BP450/50 nm; 488 nm and 525/50; 561 nm and BP595/50 nm; and 633 nm and 700/75 nm. The laser intensity was adjusted to minimize photobleaching. The images were collected using a Plan-Apochromat 60x/1.4 oil immersion objective. The detector sensitivity was adjusted for each sample to optimize the image brightness and to avoid saturation. The data was collected as images of size 1024 × 1024 pixels with a pixel size of 83 nm in x/y, and as 3D z-stacks containing 80–100 slices with 200 nm intervals.

For live cell imaging during manual or motorized relaxation, MDCK cells expressing EGFP – lamin A chromobody or H2B-EGFP were cultured on fibronectin (10 µg/mL) coated thin Si membrane assembled in Brick Strex L with a 25% pre-strain and enclosed into a base with a brick lid. Once the desired confluency was reached, the cell medium was replaced with a medium containing HEPES (25 mM) and the device was attached with metal clamps onto a pre-heated Nikon Eclipse FN1 upright microscope stage. For imaging, CoolLED pE-4000 was used as a light source (470 nm LED) to image using a pre-heated water dip objective (Nikon CFI Apo LWD 25x/1.1, WD 2.0 mm). Images of were acquired with Hamamatsu Orca-Flash4.0 V2 CMOS camera. The data was collected with a pixel size of 325 nm in x/y. When indicated, the microscope stage was moved accordingly to compensate the movement of the cell monolayer. The cell monolayer movement was always less than the size of the field of view allowing to follow the same position within the sample.

### Image analysis

The acquired data was analyzed by using ImageJ FIJI distribution. For publication figures, only linear adjustments of the microscopy image brightness and contrast were done. Microscopy images were also always filtered to determine I) the fluorescence intensities of $\upbeta$-catenin and YAP1 (Gaussian filtering, radius 1) from the nucleus and the cytoplasm in the middle optical section to calculate the nucleus to cytoplasm ratio as a measure of translocation, or II) the number of cells per field of view (Gaussian filtering, radius 2) by applying manual thresholding and the analyze particles function in ImageJ FIJI (n = 2). The nucleus-to-cytoplasm ratio of $\upbeta$-catenin and YAP1 were measured as the mean fluorescence intensity acquired from the nuclear area, segmented by the default thresholding method in ImageJ by using the DAPI stain, and from the cytoplasmic area after subtraction of the segmented nuclei from the images. For the alignment of the live cell stepwise stretching and compression data, the imaged fields were cropped so that certain nucleus was always kept in the same position. Cyclic stretching data was first manually cropped and then aligned with StackReg -plugin in FIJI. Color-coding of the data was conducted with Temporal-Color code function in ImageJ.

### Cell lysis and western blot

For western blotting, the cells were seeded in 8.8 cm^2^ cell culture column prepared by cutting from 50 mL NUNC-tube and attached with silicone grease on fibronectin (10 $\upmu$g/mL) coated thin Si membrane assembled in Brick Strex L with a 25% strain. In control studies, the cells were seeded on unstretched PDMS membranes. To detect the mechanosensitive proteins prior and after the relaxation, the cells from the control and the compressed membranes, respectively, were lysed as previously described^65^. The entire method to produce the lysates and to run the western blot can be found in Supplementary methods section online. The immunoblotting was done using anti-YAP1 (1:1000, YAP163.7, sc-101199, Santa Cruz Biotechnology), anti-$\upbeta$-actin (1:5000, ab6276, Abcam), anti-YAP1 S127P (1:1000, #13,008, Cell Signaling Technology, Denvers, MA, USA), and anti-$\upbeta$-catenin (1:1000, ab6302, Abcam) primary antibodies for o/n in + 4 °C in tilting followed incubation with a horseradish peroxidase -conjugated goat-anti mouse/rabbit secondary Abs for 1 h in RT (in tilting).

### Statistical analysis

To analyze for the differences in the nucleus to cytoplasmic localization of $\upbeta$-catenin and YAP1, and number of cells per field of view, normal distribution testing using Kolmogorov–Smirnov and independent samples unpaired Student´s t-tests were performed in GraphPad Prism version 9.1.1, GraphPad Software (San Diego, CA, USA), www.graphpad.com.

## Supplementary Information

Supplementary Information.

Supplementary Video 1.

Supplementary Video 2.

Supplementary Video 3.
