# Supplementary material for: Brick Strex: a robust device built of LEGO bricks for mechanical manipulation of cells
Source: Sci Rep. 2021 Sep 16;11:18520. doi: 10.1038/s41598-021-97900-5 (PMC8445989; doi:10.1038/s41598-021-97900-5)
Supplement: Supplementary file 1 — Supplementary Information. [file 41598_2021_97900_MOESM1_ESM.docx]

**Supplementary material**

**Brick Strex – a robust device built of LEGO bricks for mechanical manipulation of cells**

Elina Mäntylä,^a^  Teemu O. Ihalainen^a^

^a^ BioMediTech, Faculty of Medicine and Health Technology, Tampere University, Tampere, Finland

Address: BioMediTech, Faculty of Medicine and Health Technology, Tampere University, Tampere, Finland. Arvo Ylpön katu 34, 33520 Tampere, Finland

Phone: +358 50 3187 202

**Supplementary material contents:**

Supplementary Table 1: Device S part list

Supplementary Table 2: Device L part list

Supplementary Table 3: Key features of the Brick Strex devices

Supplementary Figure 1: Device S, part list images

Supplementary Figure 2: Device L, part list images

Supplementary Figure 3: Device L base, part list images

Supplementary Figure 4: Device L lid, part list images

Supplementary Figure 5: Strain measurement scale for the devices

Supplementary Figure 6: Printable strain measurement scale

Supplementary Figure 7: Effect of strained substrate on epithelial growth

Supplementary Figure 8: Full sized western blots of Yap1 and beta-catenin

Supplementary Figure 9: Changes in object density with different strains

Supplementary Figure 10: Motorization scheme of Device L

Supplementary Figure 11: Cycling stretching programming example using LEGO Mindstorms

Supplementary Methods

Supplementary Movie Titles


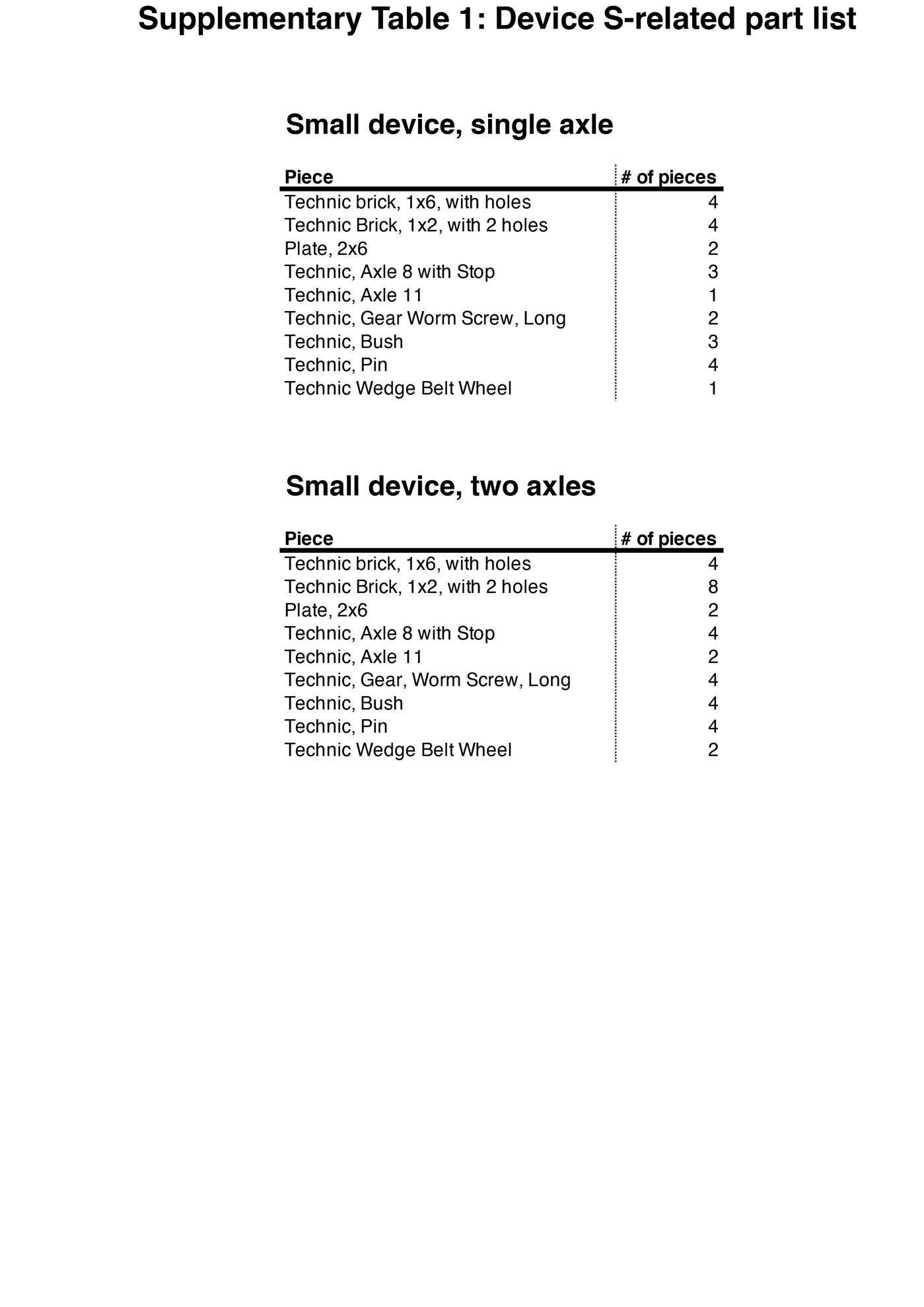


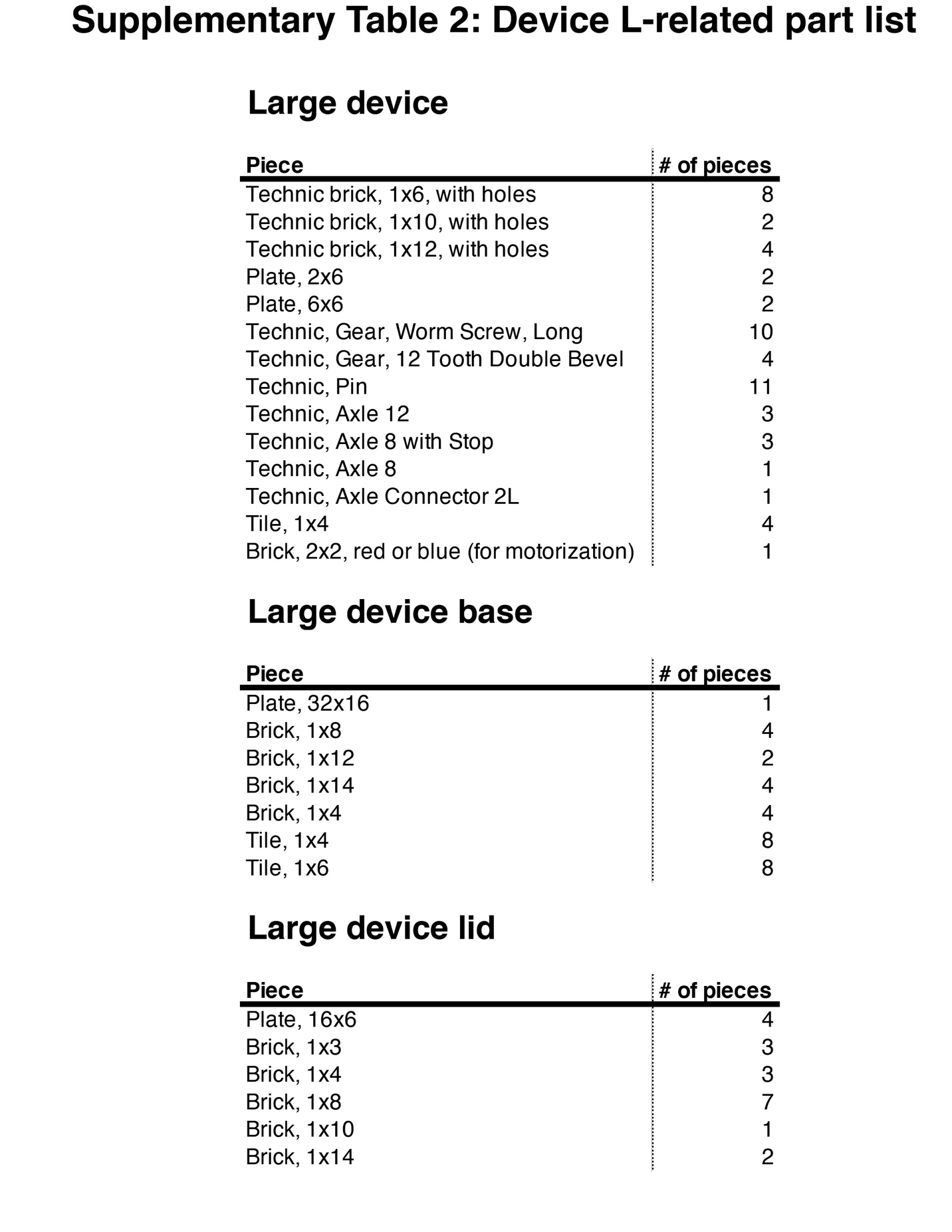


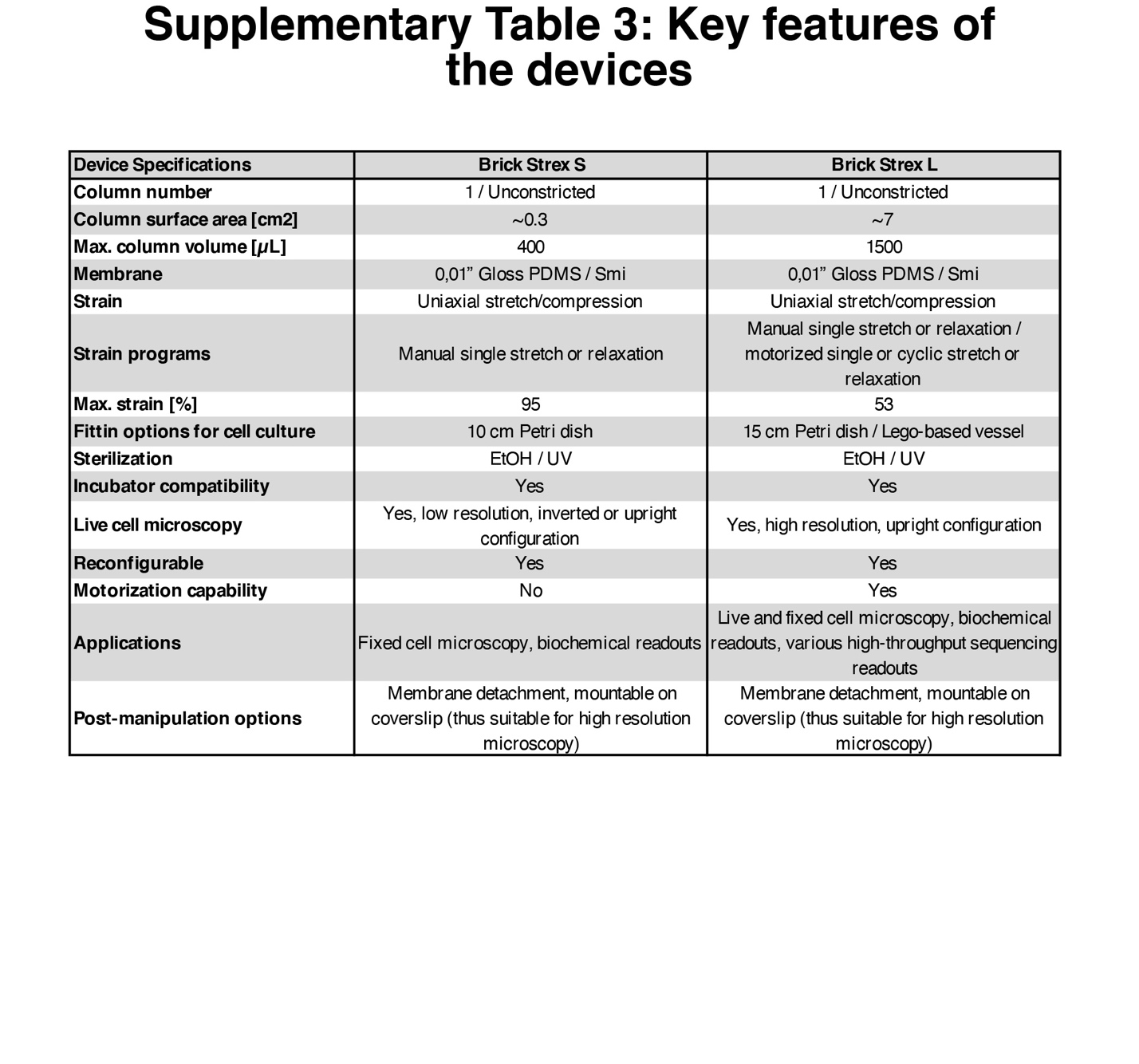


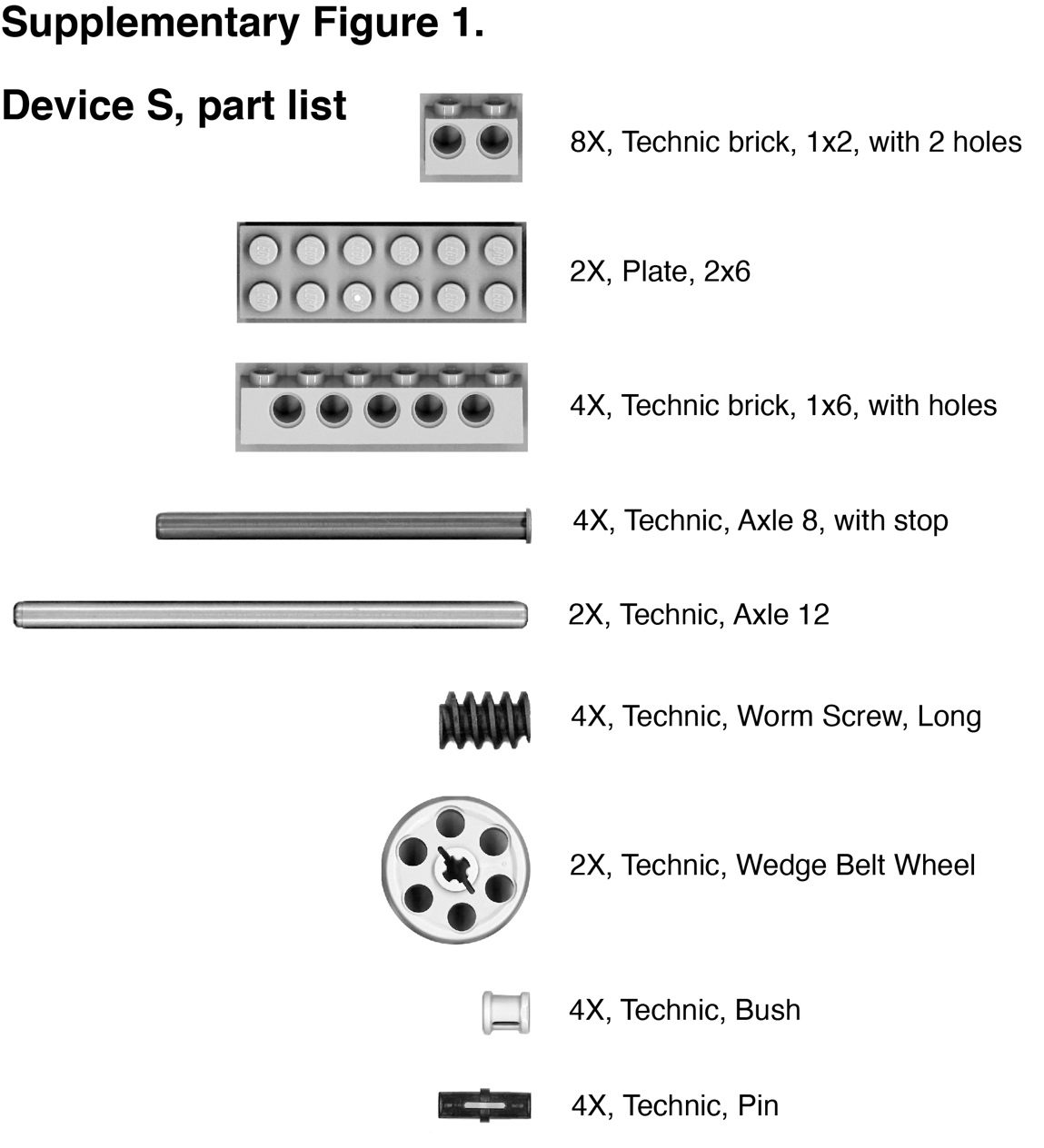


**Supplementary Figure 1: Part list of device S.** List of LEGO blocks which allow the construction of Brick Strex S device with two axles.


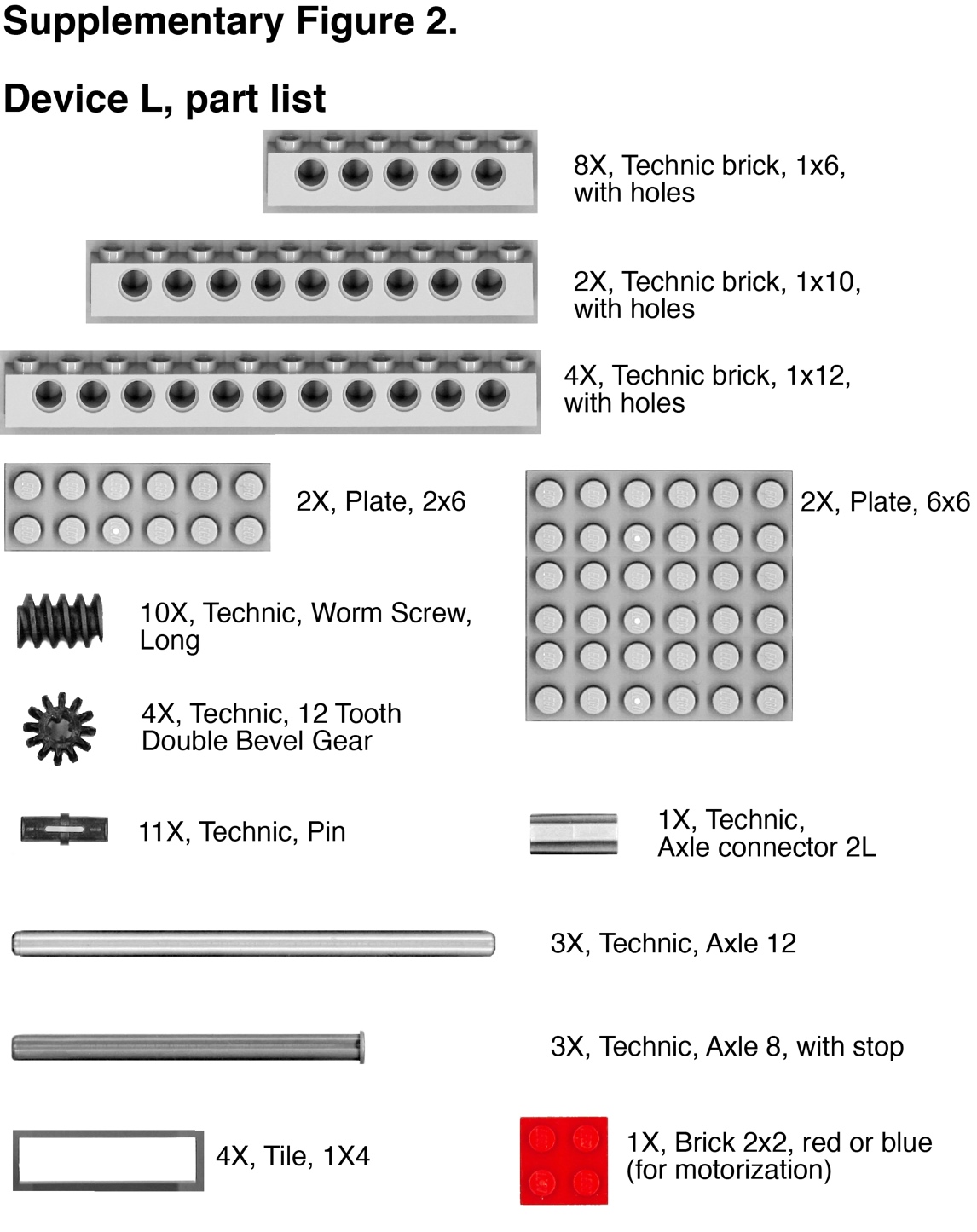


**Supplementary Figure 2: Part list of device L.** List of LEGO blocks which allow the construction of Brick Strex L device.


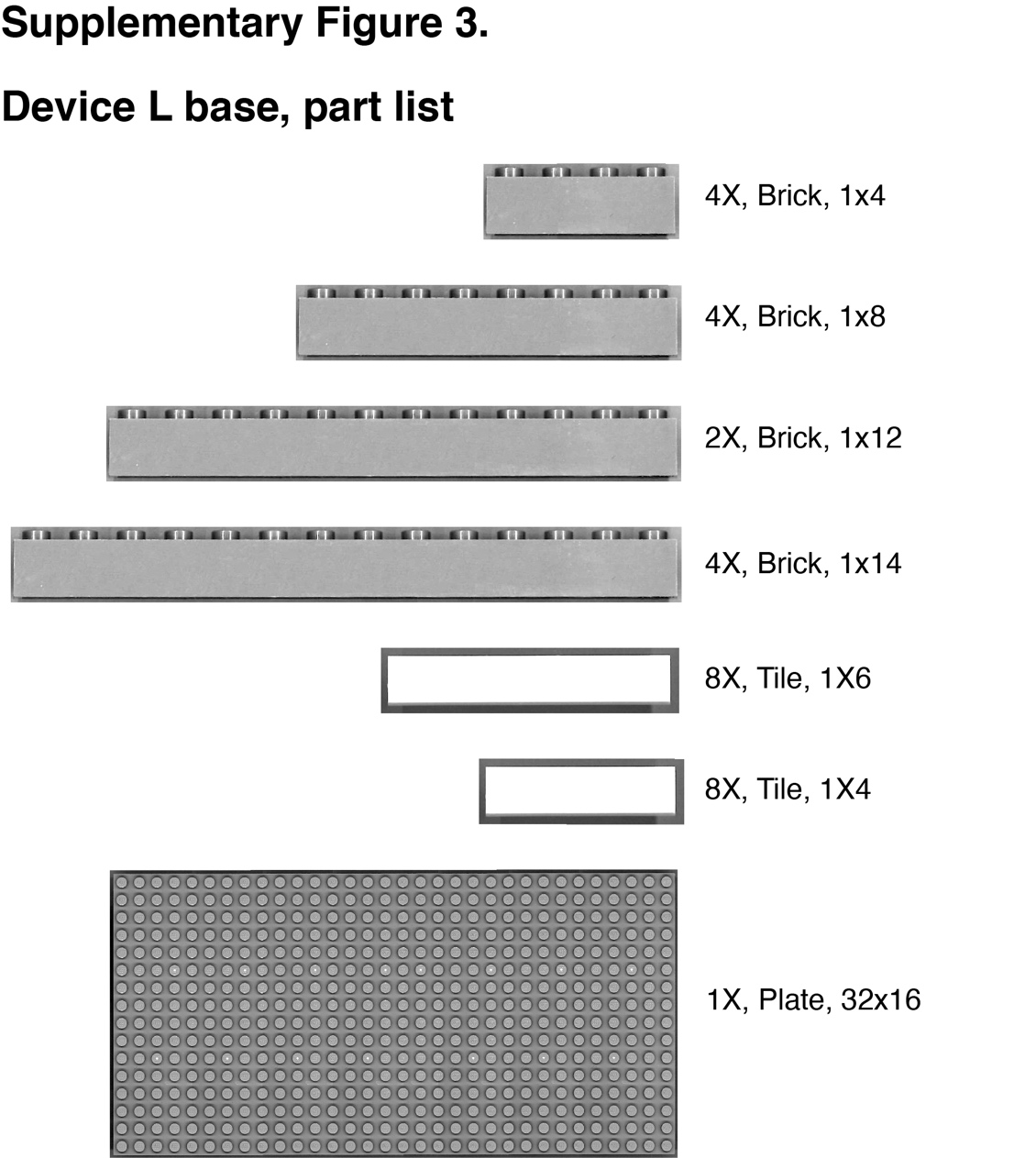


**Supplementary Figure 3: Part list of device L base.** LEGO blocks which allow the construction of Brick Strex L device base.


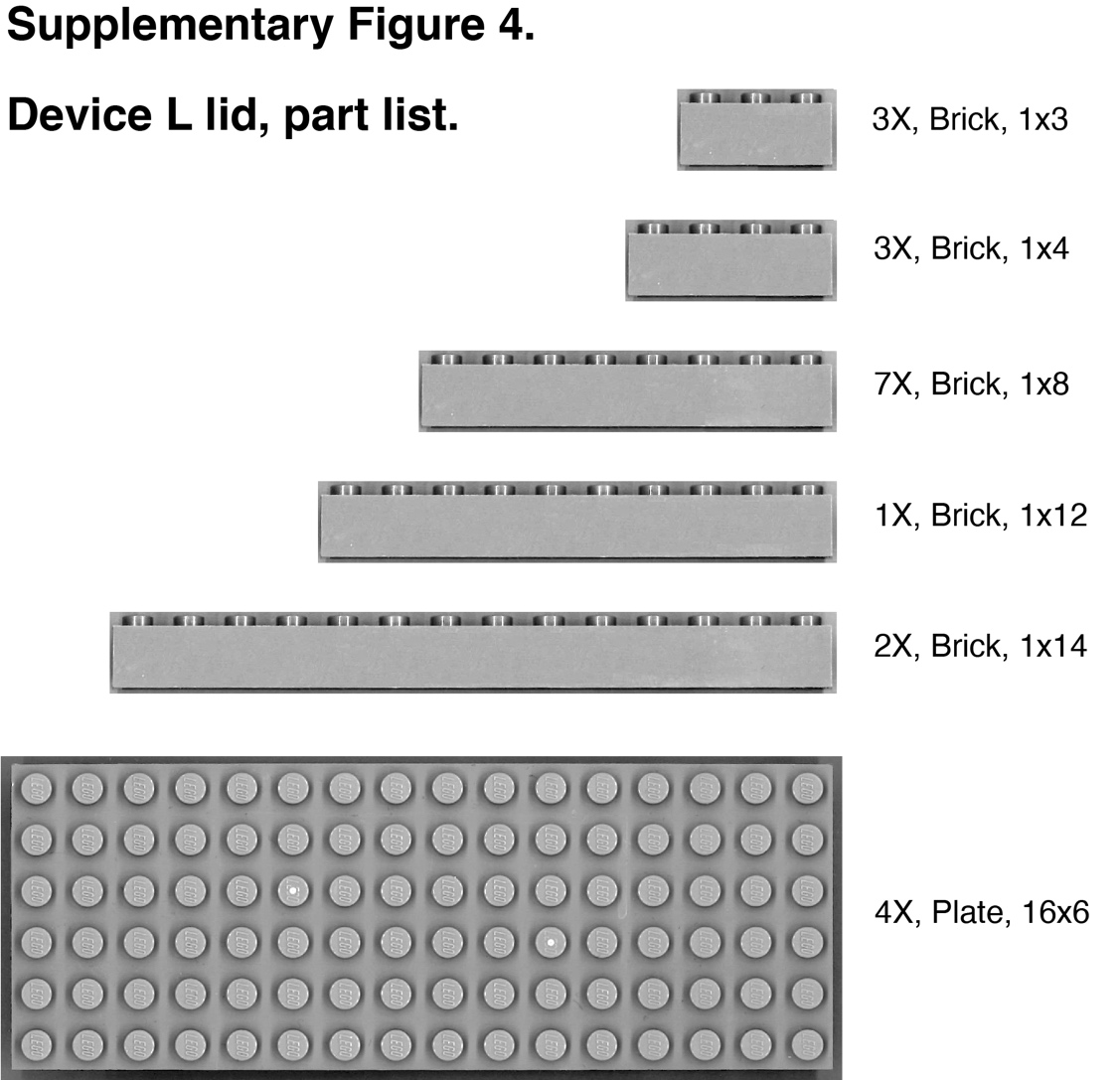


**Supplementary Figure 4: Part list of L lid.** List of LEGO blocks which allow the construction of Brick Strex L device lid.


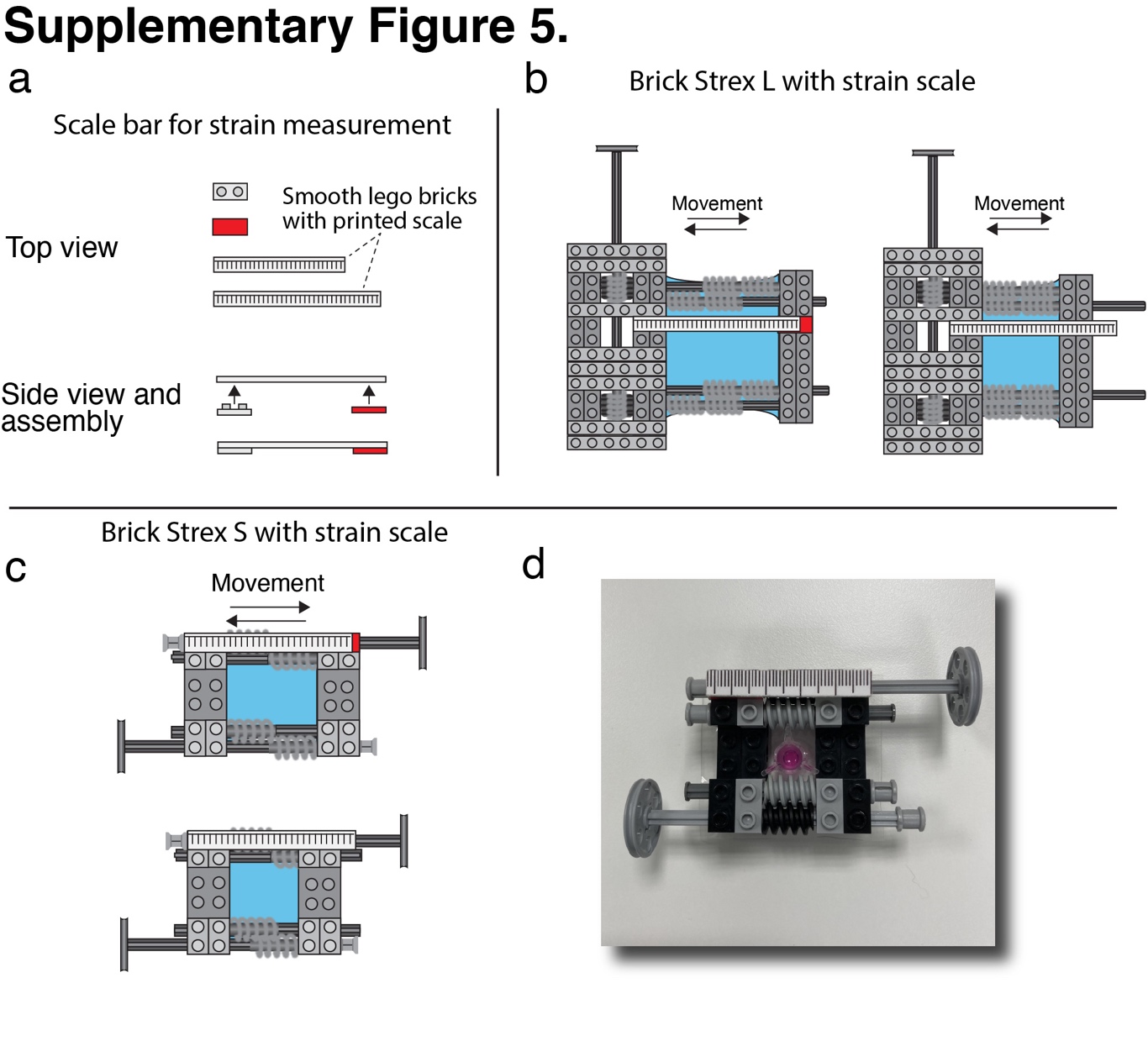


**Supplementary Figure 5: Strain measurement scale for the devices. a.** Additional bricks needed for supplementing **b**, the Brick Strex L device or **c**, the Brick Strex S with an add-on scale module allowing for direct measurement of the strain. **d**, Representative image of a Brick Strex S in an experimental setup.

**
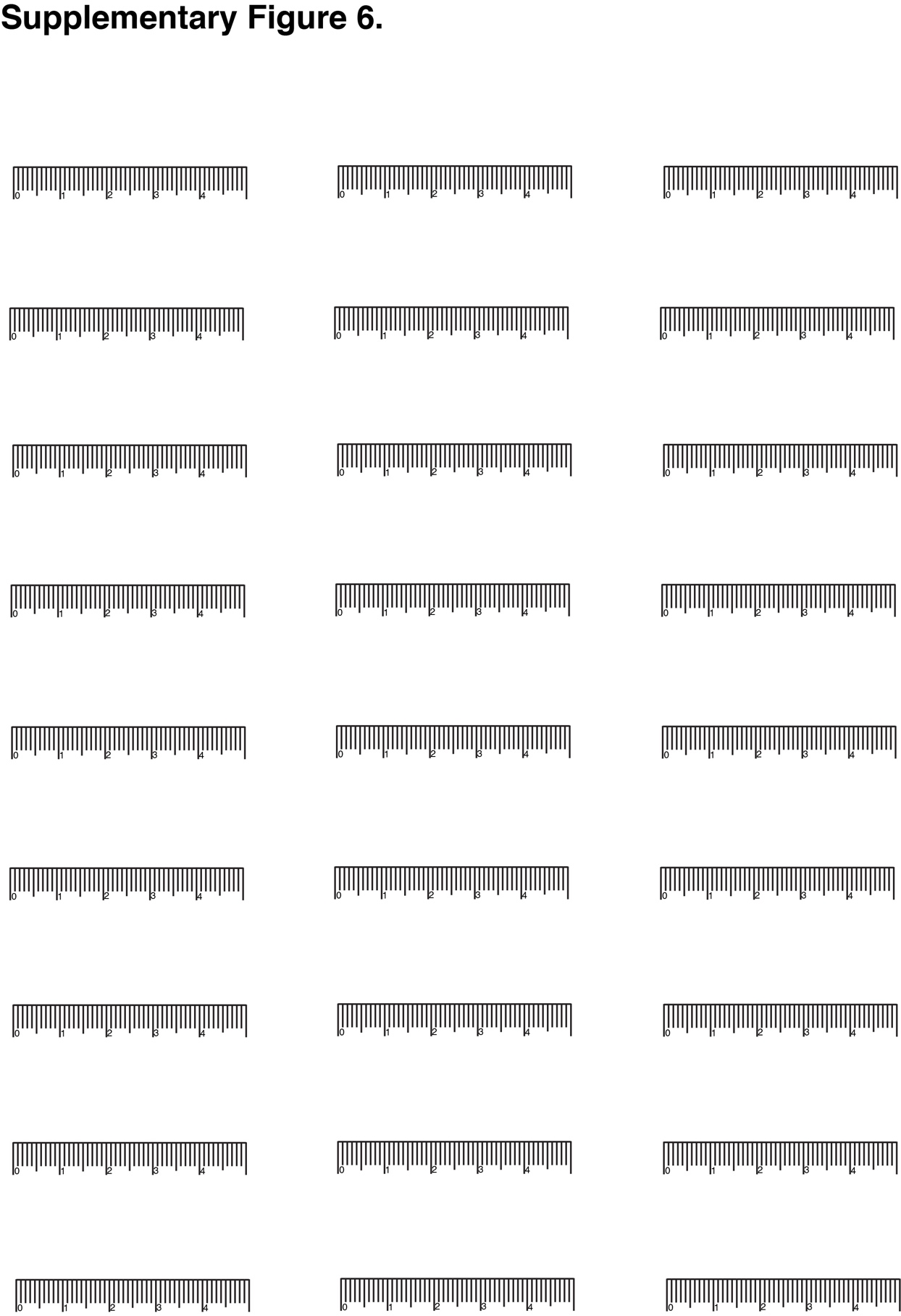
**

**Supplementary Figure 6: Printable strain measurement scale.** The printed scales can be used with Brick Strex devices with additional bricks to build an add-on scale module to continuously follow the strain. Printable to A4-sized adhesive paper.

**
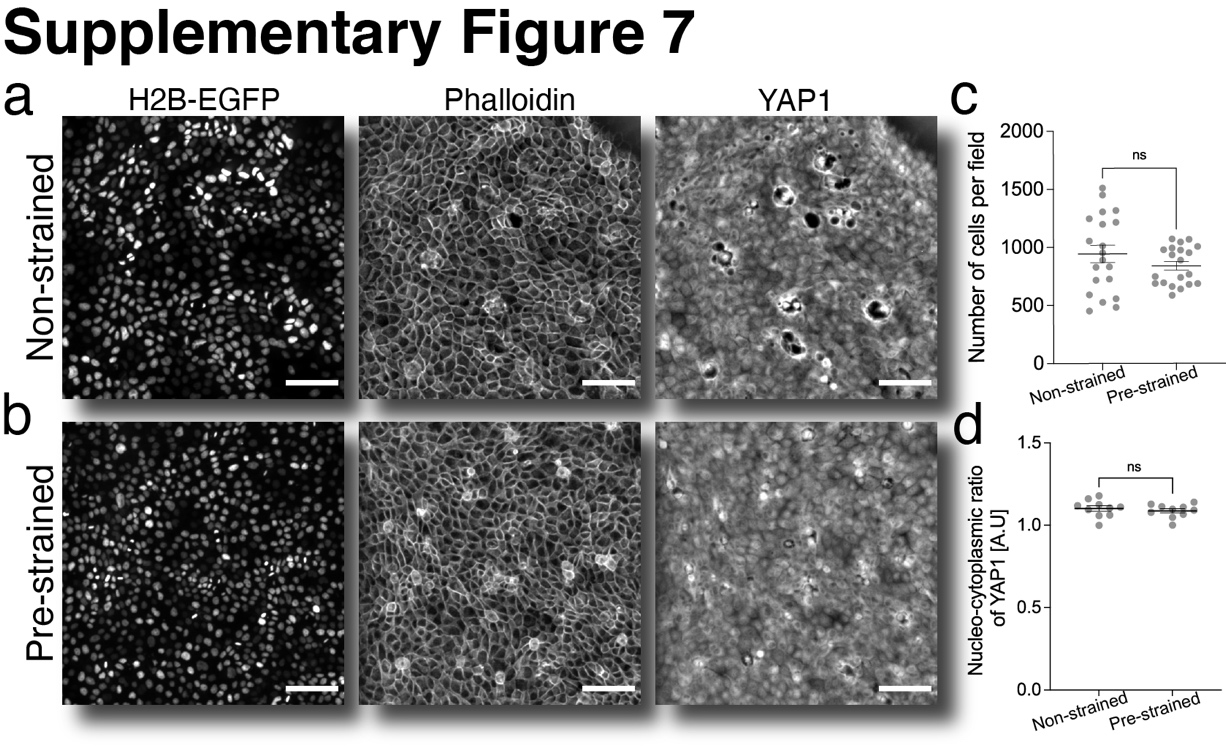
**

**Supplementary Figure 7: Effect of substrate strain on epithelial growth.** Epithelial growth on **a**, Non-strained and **b**, pre-strained (25 % strain) substrates. Nuclei are highlighted with H2B-EGFP fusion protein, actin cytoskeleton with phalloidin and YAP1 with immunolabeling. **c**, Quantification of the mean number of cells per field of view. **d**, Quantification of the nucleo-cytoplasmic ratio of YAP1. Statistical analyses were performed using an un-paired Student´s t-test when comparing between cells growing on non-strained/pre-strained substrates (ns: non-significant).

**
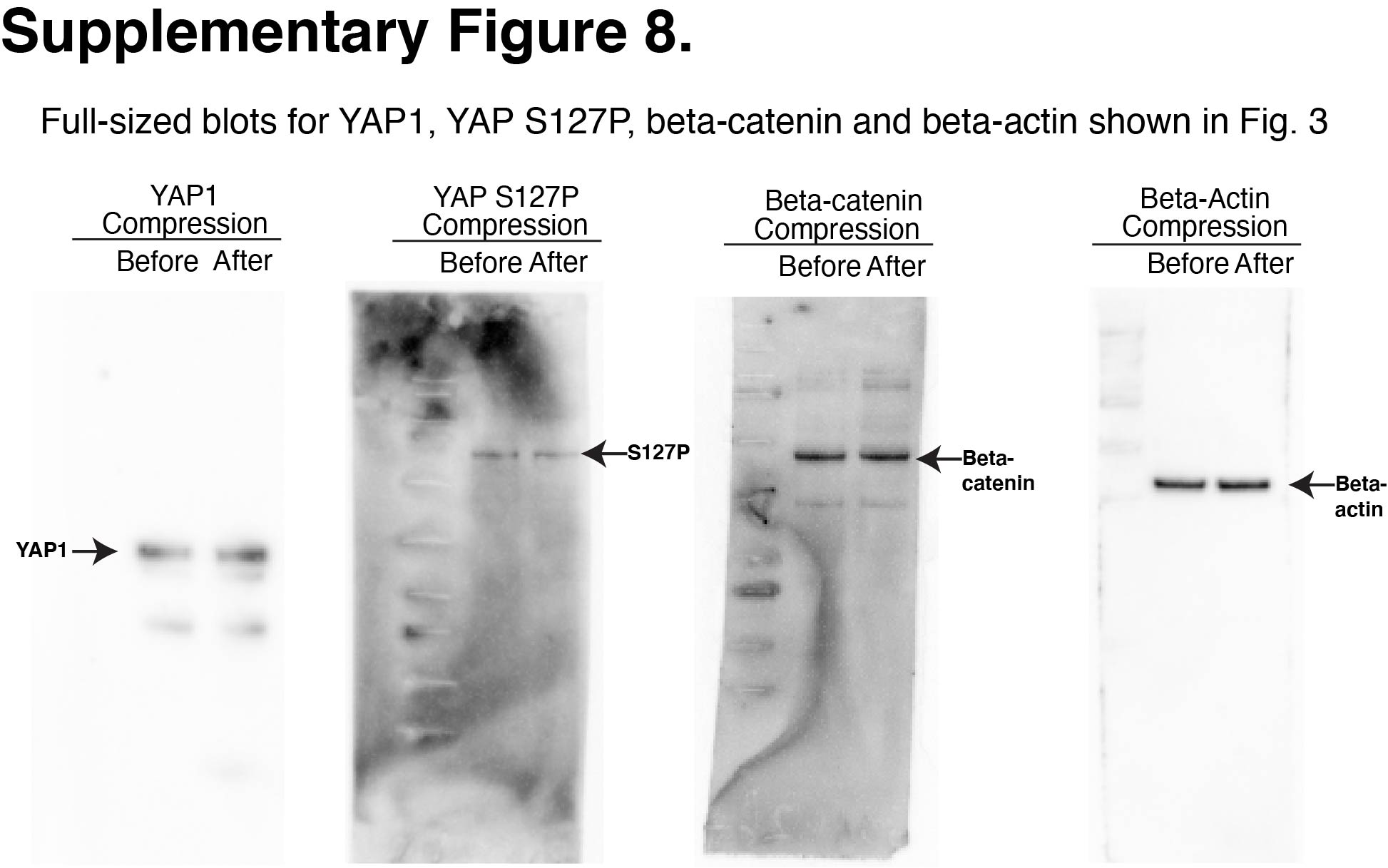
**

**Supplementary Figure 8: Full sized blots of YAP1, YAP1 phosphorylation S127P,** β**-catenin and** β**-actin.** Please see also Figure 3.

**
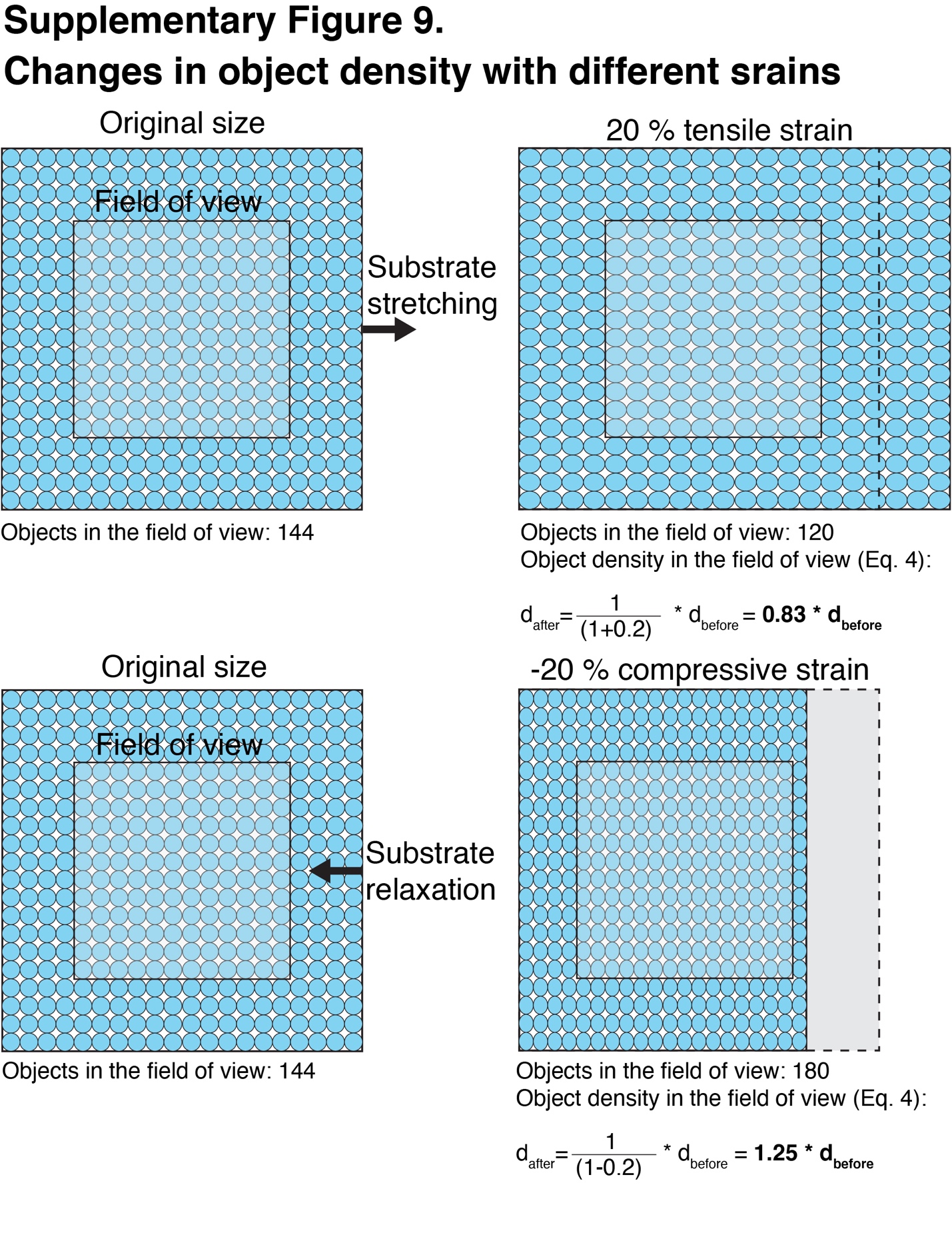
**

**Supplementary Figure 9: Changes in object density with different strains.** Visualization and quantification of changes object density as function of strain (assuming Poisson ratio = 0).


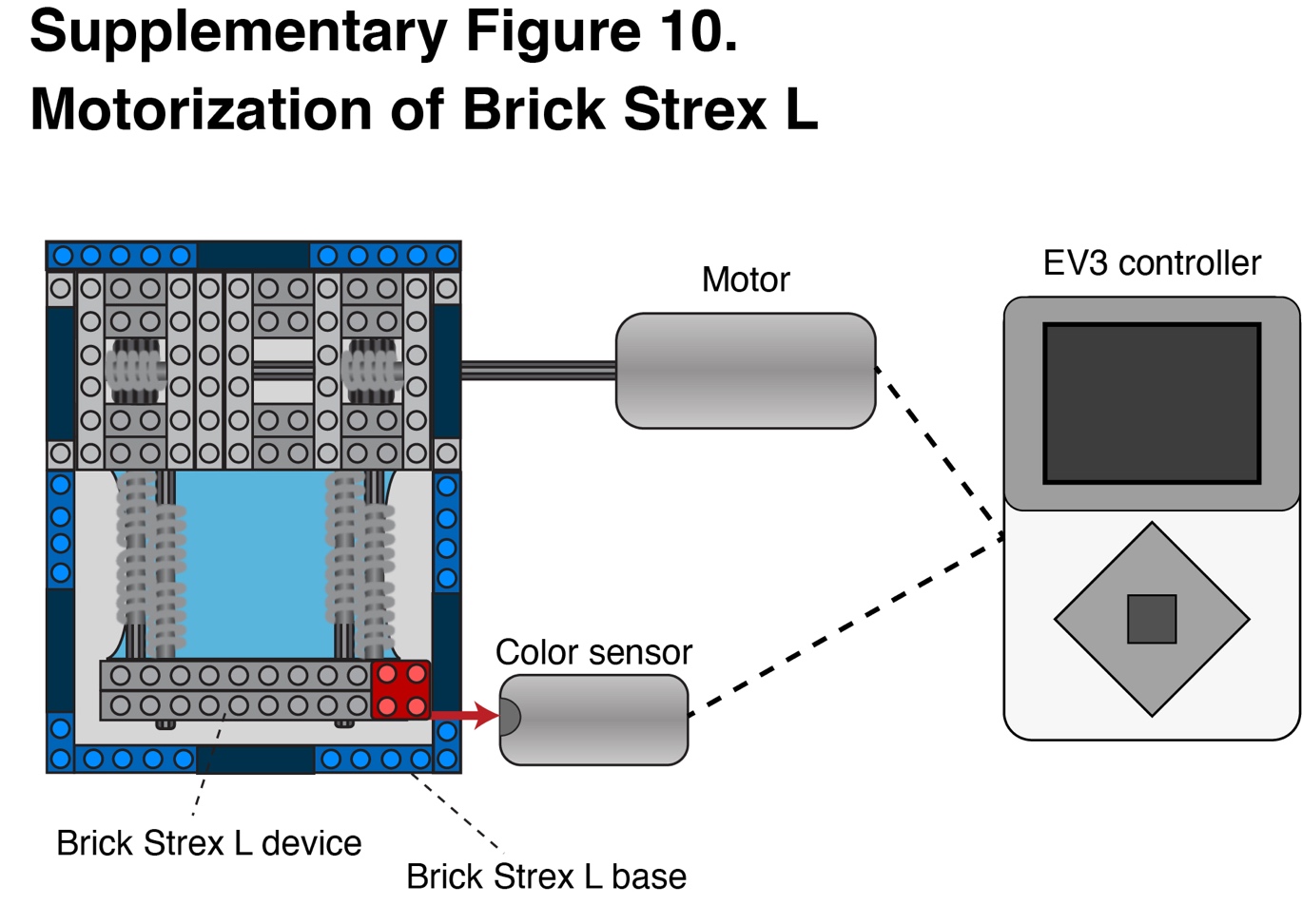


**Supplementary Figure 10: Motorization of Brick Strex L.** Automation of the stretching is achieved via motor and a color sensor, which is used to monitor and control the device movement.

**
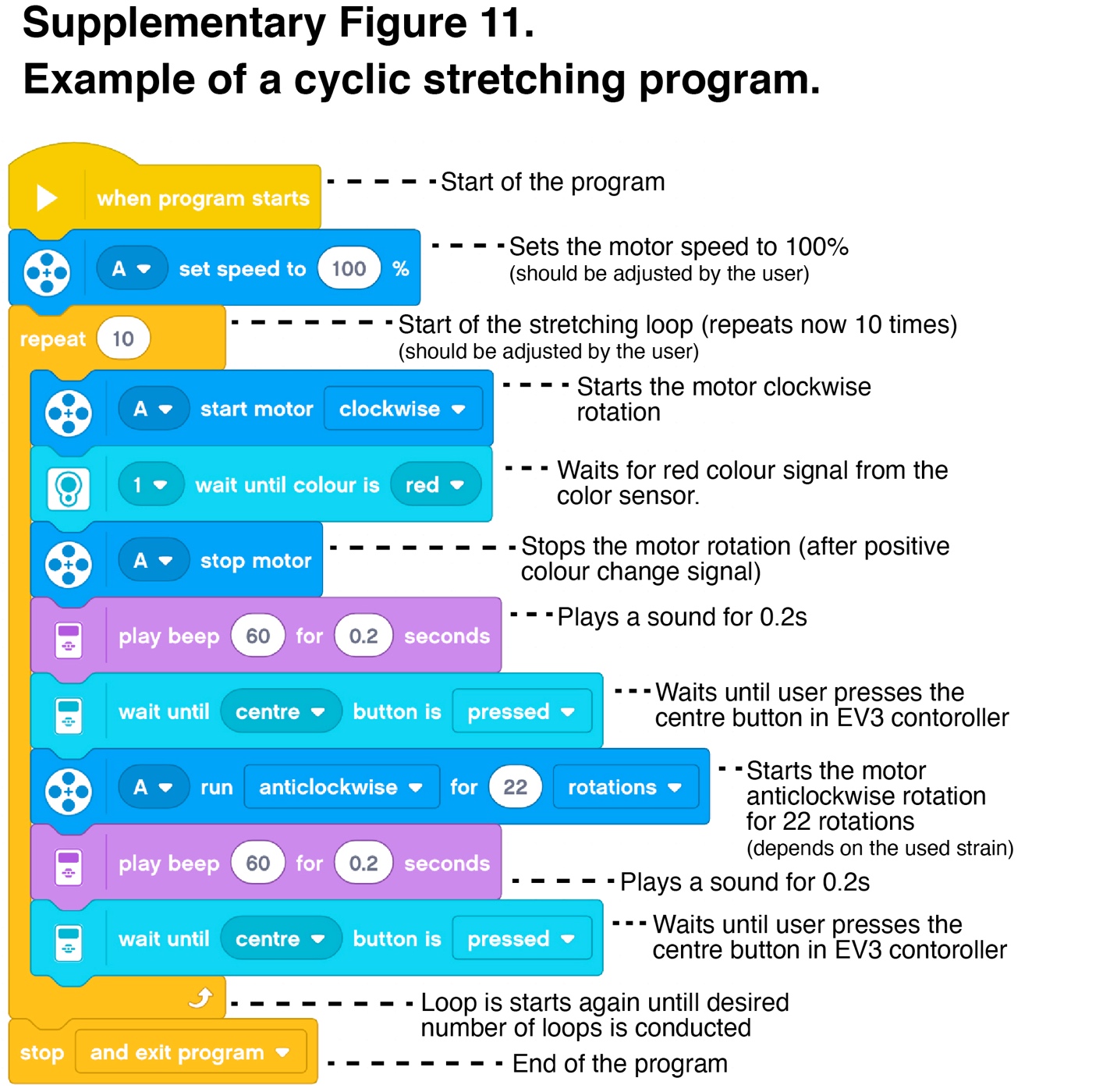
**

**Supplementary Figure 11: Programming example for cyclic stretching experiments.** Example of a LEGO Mindstorms program for cycling stretching experiments using the Brick Strex L device.

**Supplementary Methods**

**Cell Culture**

Madin-Darby canine kidney (MDCK) type II cells stably expressing histone H2B-EGFP and occludin-mEmerald were maintained in Modified Eagle´s medium (#51200046, Thermo Fisher Scientific, Waltham, MA, USA) supplemented with 1 % (vol/vol) penicillin-streptomycin antibiotics (#15140122, Thermo Fisher Scientific) and 10 % fetal bovine serum (#10500064, Thermo Fisher Scientific) under standard conditions in a humidified cell incubator (+37 °C, 5 % CO2). For the lateral compression experiments using Brick Strex S, 2 x 10^4^ cells were seeded in cell culture columns (n=2) attached onto fibronectin (10 µg/mL) -coated and UV-treated (30 min) thin (0.01") USP class VI PDMS membranes (8x4.5 cm, Specialty Manufacturing, Inc., Saginaw, MI, USA) using high-vacuum silicone grease (Dow Corning, Merck, Darmstadt, Germany). The PDMS membranes were assembled in Brick Strex with a 25 % strain (measured as 25 % increase in stretch) and/or in control experiments, prestained without stretching. The columns were prepared by cutting the column off from a PET insert. Cells were grown until a desired confluency was reached, fixed, mounted with a high-performance glass coverslip, cured o/n at RT in dark, and finally, imaged with an upright microscope.

**Immunostaining**

Cells were fixed with MetOH (ice cold, 5 min, RT), washed twice with PBS and incubated directly with a primary rabbit antibody against b-catenin (1:2000 in 3 % BSA-PBS, ab6302, Abcam, Cambridge, UK), or fixed with 4 % paraformaldehyde (10 min, RT), washed twice with PBS, permeabilized with 0.5 % Triton-X100 - 0.5 % BSA in PBS (á 10 min, RT) and treated with a mouse monoclonal antibody against YAP1 (1:500 in 3 % BSA-PBS, YAP163.7, sc-101199, Santa Cruz Biotechnology, Dallas, USA) and Alexa565-conjugated phalloidin to detect actin (1:100 in 3 % BSA-PBS, 1 h in RT, in dark). After the primary antibody incubation, the samples were washed with permeabilization buffer (0.5 % Triton-X100 - 0.5 % BSA in PBS), PBS and again with permeabilization buffer (á 10 min, RT). Next, Alexa 488-conjugated goat anti-mouse and Alexa 647-conjugated goat anti-rabbit secondary antibodies (Thermo Fisher Scientific, Waltham, MA, USA) were added and incubated for 1h (1:200 in 3 % BSA-PBS, RT, in dark). After the secondary antibody incubation, the samples were washed twice with PBS (á 10 min, RT, in dark) and rinsed once with dH_2_O. The samples were detached from the stretching device and the column was detached followed by removal of the silicon grease. Finally, 18 x 18 mm silicon piece was cut out and moved to an objective glass and mounted with ProLong Diamond Antifade Mountant with DAPI (Thermo Fisher Scientific) by placing a 22x22 mm high-performance glass coverslip (Carl Zeiss Microscopy) on top. Samples were cured in RT in dark overnight (o/n) and stored in +4°C prior to imaging.

To analyze for the effect of PDMS membrane strain on the growth, biomechanical condition and morphology of the epithelial monolayer, the MDCKII cells stably expressing H2B-EGFP were fixed with 4 % PFA for 10 min in RT, washed twice with PBS and finally, immunolabelled against actin (1:100, phalloidin, Thermo Fischer Scientific) and YAP1 (1:1000, YAP163.7, sc-101199, Santa Cruz Biotechnology) as shown in Supplementary Figure 7.

**Image analysis**

The acquired data was analyzed by using ImageJ FIJI distribution (https://imagej.net/software/fiji/). For publication supplementary figures, linear adjustment of the microscopy image brightness and contrast was done. Microscope images were filtered (gaussian filtering, radius 1 or 2) to determine the mean number of cells per field of view and the intracellular localization of YAP1. Quantification of the number of cells per field of view was done by counting the nuclei from manually thresholded images and applying the analyze particles function in ImageJ FIJI (n=2).

**Western blotting**

Lysates for western blotting were produced as described earlier^65^. Brick Strex L devices were put on ice and the medium was aspirated followed by rinse with ice-cold PBS. Next, the cells were overlaid with 0.5 mL of cell lysis buffer [100 mM KCl, 5 mM MgCl_2_, 10 mM Hepes, pH 7.0, 0.5 % NP-40, and 10 000 U/mL Halt Protease Inhibitor Cocktail 100X (#78429, Thermo Fisher Scientific)] and detached by scraping on ice. The cell lysate was next transferred into a tube pre-cooled on ice. After incubation for 10 min at +4°C on ice, the samples were centrifuged at 5000 rpm for 5 min followed by collection of the supernatants considered to be the cytoplasmic fractions while the pellets were kept as to obtain the nuclear fraction. The nuclear fraction was produced by resuspending the pellets in 250 µl of cold high salt lysis buffer [400 mM KCl, 5 mM MgCl_2_, 10 mM Hepes, pH 7.0, 0.5 % NP-40, 1 mM DTT, and 10 000 U/mL protease inhibitor cocktail]. Next, the tubes were vigorously rocked at +4 °C for 45 min on a shaking platform. The supernatants i.e. the nuclear extracts were collected after centrifugation at 10 000 × *g* for 30 min. The cytoplasmic and nucleic lysates were then pooled to produce a total lysate. Finally, the protein concentrations were measured with NanoDrop and the samples were stored in -70°C prior immunoblotting.

For immunoblotting, a total of 20 mg of protein was mixed with SDS-Laemmli 4X sample buffer and boiled for 10 min. After cooling, the samples were briefly centrifuged (10 000 × g for 1 min) and loaded on a NuPAGE 4-12 % gradient gel (Invitrogen) along with a pre-stained dual-color molecular weight marker (#161-0376, Bio-Rad, Hercules, CA, USA). The gel was run for 5 min at 90 V after which the voltage was increased to 180 V to finish the run. Proteins were transferred to a PVDF membrane for 7 min at constant current of 400 mA (Trans-Blot Turbo transfer system with PowerPac Basic power unit, Bio-Rad). After the run, the blot was blocked in 3 % BSA in Tris-buffered saline (1X TBS) for 1h at RT. Following the primary and secondary antibody incubations described in the article, the blots were washed three times with TBST and once with dH2O (á 10 min, RT, in tilting). The detection was done by applying Pierce ECL Western Blotting Substrate (Thermo Fisher Scientific) to the blot following manufacturer´s instructions and imaged using a Bio-Rad´s CCD-camera based ChemiDoc XRS+ system (Bio-Rad).

**Statistical Analysis**

To analyze for the differences in the mean number of cells per field of view, normal distribution testing using Kolmogorov-Smirnov and independent samples unpaired Student´s t-test were performed in GraphPad Prism version 9.1.1, GraphPad Software (San Diego, CA, USA), (www.graphpad.com)

**Supplementary Movies**

**Supplementary Movie 1: Assembly of Brick Strex S with one axle.**

**Supplementary Movie 2: Assembly of Brick Strex L and the base.**

**Supplementary Movie 3: Live cell microscopy of motorized single stretch experiment by using Brick Strex L.**
